# Oxygen versus air-driven nebulisers for exacerbations of chronic obstructive pulmonary disease: a randomised controlled trial

**DOI:** 10.1186/s12890-018-0720-7

**Published:** 2018-10-03

**Authors:** George Bardsley, Janine Pilcher, Steven McKinstry, Philippa Shirtcliffe, James Berry, James Fingleton, Mark Weatherall, Richard Beasley

**Affiliations:** 1 Capital and Coast District Health Board, Wellington, New Zealand; 2 Medical Research Institute of New Zealand, Box 7902, Wellington, PO 6242 New Zealand; 3 Victoria University Wellington, Wellington, New Zealand; 4 Wellington School of Medicine & Health Sciences, University of Otago Wellington, Wellington, New Zealand

**Keywords:** Air, Bronchodilator agents, Hypercapnia, Nebulisation, Oxygen

## Abstract

**Background:**

In exacerbations of chronic obstructive pulmonary disease, administration of high concentrations of oxygen may cause hypercapnia and increase mortality compared with oxygen titrated, if required, to achieve an oxygen saturation of 88–92%. Optimally titrated oxygen regimens require two components: titrated supplemental oxygen to achieve the target oxygen saturation and, if required, bronchodilators delivered by air-driven nebulisation. The effect of repeated air vs oxygen-driven bronchodilator nebulisation in acute exacerbations of chronic obstructive pulmonary disease is unknown. We aimed to compare the effects of air versus oxygen-driven bronchodilator nebulisation on arterial carbon dioxide tension in exacerbations of chronic obstructive pulmonary disease.

**Methods:**

A parallel group double-blind randomised controlled trial in 90 hospital in-patients with an acute exacerbation of COPD. Participants were randomised to receive two 2.5 mg salbutamol nebulisers, both driven by air or oxygen at 8 L/min, each delivered over 15 min with a 5 min interval in-between. The primary outcome measure was the transcutaneous partial pressure of carbon dioxide at the end of the second nebulisation (35 min). The primary analysis used a mixed linear model with fixed effects of the baseline PtCO_2_, time, the randomised intervention, and a time by intervention interaction term; to estimate the difference between randomised treatments at 35 min. Analysis was by intention-to-treat.

**Results:**

Oxygen-driven nebulisation was terminated in one participant after 27 min when the PtCO_2_ rose by > 10 mmHg, a predefined safety criterion. The mean (standard deviation) change in PtCO_2_ at 35 min was 3.4 (1.9) mmHg and 0.1 (1.4) mmHg in the oxygen and air groups respectively, difference (95% confidence interval) 3.3 mmHg (2.7 to 3.9), *p* < 0.001. The proportion of patients with a PtCO_2_ change ≥4 mmHg during the intervention was 18/45 (40%) and 0/44 (0%) for oxygen and air groups respectively.

**Conclusions:**

Oxygen-driven nebulisation leads to an increase in PtCO_2_ in exacerbations of COPD. We propose that air-driven bronchodilator nebulisation is preferable to oxygen-driven nebulisation in exacerbations of COPD.

**Trial registration:**

Australian New Zealand Clinical Trials Registry number ACTRN12615000389505. Registration confirmed on 28/4/15.

**Electronic supplementary material:**

The online version of this article (10.1186/s12890-018-0720-7) contains supplementary material, which is available to authorized users.

## Background

In acute exacerbations of chronic obstructive pulmonary disease (AECOPD), administration of high concentration oxygen may cause profound hypercapnia and increase mortality, compared with oxygen titrated to achieve an oxygen saturation of between 88 to 92% [1, 2]. Titrated oxygen regimens require two components: titrated supplemental oxygen to achieve a particular target arterial oxygen saturation measured by pulse oximetry (SpO_2_), and bronchodilators delivered by either air-driven nebulisation or metered-dose inhalers with a spacer. Oxygen-driven nebulisation inadvertently exposes patients to high concentrations of inspired oxygen, particularly with prolonged or repeated use as may occur in patients with severe exacerbations during long pre-hospital transfers or if the mask is inadvertently left in place.

We have shown that air-driven bronchodilator nebulisation prevents the increase in arterial partial pressure of carbon dioxide (PaCO_2_) that results from use of oxygen-driven nebulisers in patients with stable COPD [3]. However, there are only two small non-blinded randomised controlled trials of air compared to oxygen-driven nebulisation in patients admitted to hospital with AECOPD [4, 5]. These trials reported that administration of a single bronchodilator dose using oxygen-driven nebulisation increases the PaCO_2_ in COPD patients who have baseline hypercapnia.

Robust determination of the risks of oxygen-driven nebulisation in AECOPD could identify whether widespread implementation of air-driven nebulisers, or use of metered-dose inhalers through a spacer, are required to ensure safe delivery of bronchodilators to this high-risk patient group. The objective of this study was to compare the effects on PaCO_2_ of air- and oxygen-driven bronchodilator nebulisation in AECOPD. Our hypothesis was that two doses of oxygen-driven bronchodilator nebulisation would increase the PaCO_2_ compared with air-driven nebulisation in patients hospitalised with an AECOPD.

## Methods

### Trial design and patients

This was a parallel-group double-blind randomised controlled trial at Wellington Regional Hospital, New Zealand. The full study protocol is available in the online supplement.

Participants were hospital inpatients, ≥40 years of age, with an admission diagnosis of AECOPD. Exclusion criteria included requirement for ≥4 L/min of oxygen via nasal cannulae to maintain SpO_2_ between 88 to 92%; current requirement for non-invasive ventilation (NIV); baseline transcutaneous partial pressure of carbon dioxide (PtCO_2_) > 60 mmHg; inability to provide written informed consent; and any other condition which at the Investigator’s discretion, was believed may present a safety risk or impact on the feasibility of the study results. Written informed consent was obtained before any study-specific procedures. The study was undertaken on the ward during the hospital admission. Ethics approval was obtained from the Health and Disability Ethics Committee, New Zealand (Reference 14/NTB/200). The full study protocol (original and updated version) can be found on the OLS (see Additional file 1 and 2).

### Intervention

After written consent, participants had continuous PtCO_2_ and heart rate monitoring using the SenTec® (SenTec AG, Switzerland) device and oxygen saturation (SpO_2_) measured by pulse oximetry (Novametrix 512, Respironics, Carlsbad, USA). Participants were randomised to receive two nebulisations, both driven either by air or oxygen, at 8 L/min, each delivered over 15 min with a five minute break in-between. Randomisation was 1:1 by a block randomised computer generated sequence (block size six), provided in sealed opaque envelopes by the study statistician who was independent of recruitment and assessment of participants.

The participants and blinded investigator, who recorded heart rate and PtCO_2_ were masked to the randomised treatments. If both oxygen and air ports were available in hospital on the wall behind the participant, these were used for driving nebulisation. If only oxygen ports were available, identical portable oxygen and air cylinders were placed behind the participant’s bed prior to randomisation and used instead. Both the participant and blinded investigator faced forward for the full duration of the study. In addition, the blinded investigator sat towards the end of the bed - ahead of the participant, such that they could not see the participant’s interventions. Likewise, the blinded investigator and patient could not view the SpO_2_ on the Sentec device, as this was covered during the interventions, or the pulse oximeter which could only be viewed by the unblinded investigator. Interaction between blinded and unblinded investigators would only occur if a rise in PtCO_2_ of ≥10 mmHg was demonstrated (a predefined safety criterion to abort intervention).

An initial 15 min wash-in and titration period was administered by the unblinded investigator using nasal cannulae, if required, to ensure that participant’s SpO_2_ were within 88 to 92%. If saturations were ≥ 88% on room air, no supplemental oxygen was required. Randomisation was performed after the 15 min wash-in period, when both patient and blinded investigator were already in a forward-facing position to maintain blinding. The unblinded investigator recorded SpO_2_ on a separate pulse-oximeter from then onwards.

Immediately before the first nebulisation, denoted by the baseline reading at time-point zero, PtCO_2_, SpO_2_ and heart rate were recorded. Participants then received two administrations of 2.5 mg salbutamol by nebulisation, delivered by either air or oxygen - each for 15 min duration at a flow rate of 8 L/min. Hudson RCI Micro Mist Nebuliser Masks (Hudson RCI, Durham, North Carolina, USA) were used. The nebulisations were delivered by the unblinded investigator at time zero and at 20 min, allowing for a five minute interval between nebulisations. Recordings were continued for 45 min after completion of the last nebulisation (80 min after baseline). Measurements of PtCO_2_, SpO_2_ and heart rate were recorded at five minute intervals, and at six minutes after the start of each nebulisation, in view of the British Thoracic Society (BTS) guideline’s recommendation for limiting oxygen-driven nebulisation to six-minutes in ambulance care, if air-driven nebulisation is unavailable [6].

Immediately before the first nebulisation and just before completion of the second nebulisation, at 35 min, a capillary blood gas sample was taken from the fingertip for measurement of PcapCO_2_ and pH.

### Oxygen delivery

During the wash-in and between the nebulisations oxygen was titrated, if required, via nasal prongs to maintain oxygen saturations between 88 to 92%. Participants in the air-driven group who were receiving oxygen at the start of nebulisation continued to receive titrated supplemental oxygen via nasal prongs underneath the nebuliser mask. Those in the oxygen-driven group had the prongs removed at the start, and reapplied after the completion of each nebulisation. At 35 min, oxygen was delivered via nasal prongs to participants at the flow rate they last received during titration (i.e. at 35 min and 20 min in the air-driven and oxygen-driven groups, respectively). From 35 min until 80 min, the oxygen flow rate was only increased (or initiated) if a participant’s SpO_2_ fell below 85%.

### Outcomes

The primary outcome was originally planned to be PcapCO_2_ at 35 min, at completion of the second nebulisation. However, after the first 14 participants had been studied, it was evident that obtaining adequate amounts of blood to fill the capillary tubes from some participants was difficult. At this stage of recruitment 4/14 (29%) of participants had missing data. The primary outcome variable was therefore changed to PtCO_2_ at 35 min, with PcapCO_2_ at 35 min reverting to a secondary outcome variable. Other secondary outcomes were the individual PtCO_2_ measurements at each time point; the proportion of participants who had a rise in PtCO_2_ or PcapCO_2_ of ≥4 and ≥ 8 mmHg; capillary pH at 35 min, and heart rate and SpO_2_ measurements at each time point.

### Sample size calculation and statistical analysis

A rise in PtCO_2_ from baseline of ≥4 mmHg is considered a physiologically significant change and ≥ 8 mmHg a clinically significant change, based on previous criteria [7, 8]. In our study of oxygen versus air-driven nebulisers in stable COPD patients, the standard deviation (SD) of baseline PtCO_2_ was 5.5 mmHg [3]. With 90% power and alpha of 5%, 82 patients were required to detect a 4 mmHg difference. Assuming a drop-out rate of 10% our target recruitment was 90 patients.

The primary analysis used a mixed linear model with fixed effects of the baseline PtCO_2_, time, the randomised intervention, and a time by intervention interaction term; to estimate the difference between randomised treatments at 35 min. A power exponential in time correlation structure was used for the repeated measurements. The secondary outcome variables of PtCO_2_ at the other time points, SpO_2_ and heart rate used similar mixed linear models. PcapCO_2_ and pH were compared by Analysis of Covariance with the baseline measurement as a continuous co-variate. As a post-hoc analysis we compared the difference in PtCO_2_ between the 15 and 6 min, and the 35 and 26 min time points.

Comparison of categorical variables, PtCO_2_ or PcapCO_2_ change of ≥4 and 8 mmHg, was by estimation of a risk difference, and Fishers’ exact test. As a post-hoc analysis we also compared the difference in paired proportions for those with PtCO_2_ change of ≥4 mmHg in the oxygen arm only using McNemar’s test and an appropriate estimate for the difference in paired proportions. The time for PtCO_2_ to return to baseline during the observation period (defined as the time until the PtCO_2_ was first equal to or below the baseline value, between 40 and 80 min), was compared using Kaplan-Meier survival curves and a Cox Proportional Hazards model. A simple t-test was used to compare the lowest value of the SpO_2_ between 40 and 80 min, compared to baseline. SAS version 9.4 was used.

## Results

### Patients

**Fig. 1:**
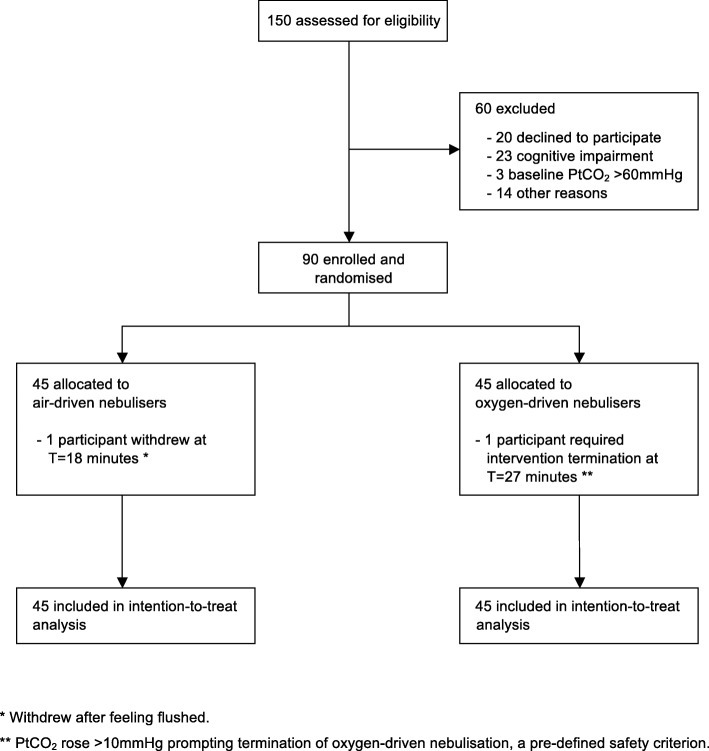
Participant flow through the study and allocation of interventions

The trial recruited between May 14th 2015 and June 29th 2016. The CONSORT diagram of the flow of the 90 recruited participants through the trial is shown in Fig. 1. One participant withdrew after 18 min of air-driven nebulisation because of feeling flushed, and so complete data was available for PtCO_2_ for 89 participants. The baseline PtCO_2_ for this participant was 34.3 mmHg and at the time of withdrawal it was 34.6 mmHg. Oxygen-driven nebulisation was stopped in another participant at 27 min when the PtCO_2_ rose by > 10 mmHg from baseline, a pre-defined safety criterion. The baseline PtCO_2_ for this participant was 43.4 mmHg and at the time of withdrawal it was 54.1 mmHg. This participant had study measurements continued after this for the full duration of the study. No clinical adverse events were noted during the intervention periods.

A summary of baseline participant characteristics are shown in Table 1. Participants predominantly had severe airflow obstruction with a mean FEV_1_ of 34.5% predicted. The mean (range) baseline PtCO_2_ was 37.6 mmHg (24.3 to 58.5 mmHg), and mean SpO_2_ was 93%. Patients randomised to the oxygen group were more likely to have required assisted ventilation previously. The mean (SD) time for the nebulised salbutamol to dissipate from the chamber was 5.2 (1.2) minutes.

**Table 1:** Participant Characteristics

	Mean (SD)		*P*
	Oxygen *N*=45^a^	Air *N*=45^a^	
Age (years)	70·4 (10·3)	72·3 (8·3)	0.34
Age at diagnosis of COPD (years)	58·6 (12·1) *N* = 40	58·8 (12·2) *N* = 44	0.92
BMI (kg/m^2^)	27·2 (7·7)	25·5 (8·9)	0.33
Smoking pack years	39·3 (31·1)	51·2 (39·2)	0.11
FEV_1_ (L)	0·81 (0·33) *N* = 35	0·85 (0·31) *N* = 37	0.69
FEV_1_% predicted	35·0 (11·5) *N* = 35	34·0 (11·8) *N* = 37	0.73
mMRC	2·38 (1·09)	2·33 (1·04)	0.84
Baseline Transcutaneous Data			
PtCO_2_ (mmHg)	38·0 (7·7)	37·2 (6·8)	0.59
SpO_2_ (%)	92·6 (2·4)	92·6 (2·3)	0.93
Heart Rate (per minute)	89·6 (15·7)	87·0 (16·0)	0.89
Baseline capillary blood gas			
pH	7·42 (0·04) *N* = 43	7·44 (0·03) *N* = 41	0.11
PcapCO_2_ (mmHg)	40·2 (7·0) *N* = 43	38·5 (5·9) *N* = 41	0.23
	N/45 (%)		*P*
	Oxygen	Air	
Male	17 (38)	24 (53)	0.20
Ethnicity			0.49
European	24 (53)	31 (69)	
Māori	7 (16)	4 (9)	
Pacific	5 (11)	4 (9)	
Other	9 (20)	6 (13)	
Previous Ventilation (ever)	12 (27)	3 (7)	0.02
Previous Ventilation Type			0.03
NIV	10 (22)	3 (7)	
Intubation	2 (4)	0 (0)	
Previous hypercapnia	23 (51)	17 (38)	0.29
Home Oxygen	2 (4)	1 (2)	0.99
Home Nebulisers	5 (11)	12 (27)	0.10
Comorbidities			
Heart Failure	8 (18)	3 (7)	0.20
Asthma	6 (13)	2 (4)	0.27
Bronchiectasis	3 (7)	4 (9)	0.99

*COPD* Chronic Obstructive Pulmonary Disease, *BMI* Body Mass Index, *FEV*_*1*_ Forced Expiratory Volume in 1 s at time of randomisation, *mMRC* Modified Medical Research Council dyspnea scale, *PtCO*_*2*_ Transcutaneous partial pressure of carbon dioxide, *SpO*_*2*_ peripheral oxygen saturation, *PcapCO*_*2*_ Capillary partial pressure of carbon dioxide, *NIV* non-invasive ventilation

^a^Unless indicated

### PtCO_2_

**Fig. 2:**
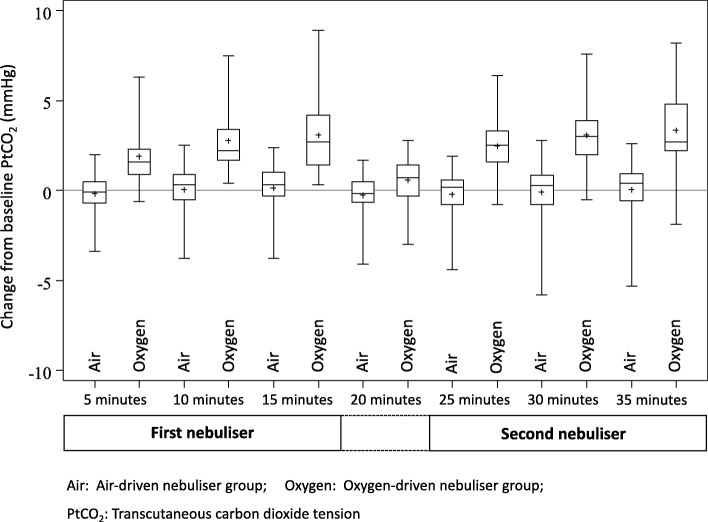
PtCO_2_ change from baseline (*T* = 0) to *T* = 35 min. Mean PtCO_2_ with error bars showing one SD, by time and intervention

The mean (SD) change in PtCO_2_ after 35 min was 3.4 (1.9) mmHg in the oxygen group (*n* = 45), compared to 0.1 (1.4) mmHg in the air group (*n* = 44). The difference (95% CI) in PtCO_2_ for oxygen compared to air-driven nebulisations after 35 min was 3.3 mmHg (2.7 to 3.9), *p* < 0.001. (Table 2 and Fig. 2). After adjustment for baseline PtCO_2_, a history of assisted ventilation, previous hypercapnia and baseline SpO_2_, were not associated with the PtCO_2_ at 35 min in either randomised group.

**Table 2:** PtCO_2_ by time and randomised group

Action	Time	PtCO_2_ Mean (SD)		Oxygen minus air	*P*
		[*N* = 45 for each unless specified]		(95% CI)	
		Oxygen	Air		
Baseline	0	38·0 (7·7)	37·2 (6·8)		
1st nebulisation	5	39·9 (8·3)	37·0 (7·1)	2·10 (1·49 to 2·71)	< 0·001
	6	40·1 (8·4)	37·0 (7·1)	2·24 (1·63 to 2·86)	< 0·001
	10	40·8 (8·6)	37·2 (6·9)	2·76 (2·15 to 3·37)	< 0·001
	15	41·1 (8·8)	37·3 (6·9)	2·97 (2·36 to 3·59)	< 0·001
	20	38·6 (7·8)	37·0 (6·4)^a^	0·86 (0·25 to 1·48)	0·006
2nd nebulisation	25	40·5 (8·3)	36·8 (6·8)^b^	2·77 (2·15 to 3·39)	< 0·001
	26	40·6 (8·4)	36·8 (6·8)^b^	2·88 (2·26 to 3·50)	< 0·001
	30	41·1 (8·5)	37·1 (6·6)^a^	3·20 (2·59 to 3·82)	< 0·001
	35	41·3 (8·6)	37·3 (6·5)^a^	3·31 (2·70 to 3·93)	< 0·001
Observation period	40	39·0 (8·1)^a^	37·0 (6·7)^a^	1·14 (0·52 to 1·76)	< 0·001
	45	38·1 (7·5)	36·7 (6·3)^a^	0·61 (− 0·01 to 1·22)	0·053
	50	37·9 (7·4)	36·6 (6·2)^a^	0·59 (− 0·02 to 1·21)	0·059
	55	37·9 (7·3)	36·6 (6·0)^a^	0·51 (− 0·10 to 1·13)	0·1
	60	37·9 (7·3)	36·7 (6·1)^a^	0·51 (− 0·10 to 1·13)	0·1
	65	38·1 (7·2)	36·7 (6·1)^a^	0·63 (0·01 to 1·25)	0·045
	70	37·5 (6·5)^a^	36·7 (6·1)^a^	0·60 (− 0·02 to 1·21)	0·059
	75	37·8 (6·8)	36·7 (6·1)^a^	0·42 (− 0·20 to 1·03)	0·18
	80	37·9 (6·9)	36·7 (6·3)^a^	0·40 (− 0·21 to 1·02)	0·2

*Air* Air-driven nebuliser group, *Oxygen* Oxygen-driven nebuliser group, *PtCO*_*2*_ Transcutaneous partial pressure of carbon dioxide

^a^*N* = 44

^b^*N* = 43

In 18/45 (40%) participants receiving oxygen-driven nebulisation, PtCO_2_ increased from baseline by ≥4 mmHg at some stage during the intervention compared to none of the participants receiving air-driven nebulisation, risk difference (95% CI) 40% (25.7 to 54.3), p < 0.001. The full data description and comparisons at each time point are shown in the OLS. Two participants receiving oxygen-driven nebulisation had a rise in PtCO_2_ ≥ 8 mmHg, one of whom required intervention termination, exceeding the predefined safety criterion of a rise ≥10 mmHg from baseline.

The estimate (95% CI) of the time-related difference, 15 min minus six minutes, for oxygen compared to air, was 0.73 mmHg (0.11 to 1.35), *P* = 0.021; and for 35 min minus 26 min, 0.43 mmHg (− 0.19 to 1.06), *P* = 0.17. In the oxygen treatment arm the proportion of patients in whom the PtCO_2_ increased from baseline by ≥4 mmHg at 6 min was less than the proportion at 15 min: 6/45 (13.3%) and 13/45 (28.9%) respectively, paired difference in proportions (95% CI) 15.6% (3.3 to 27.8), *P* = 0.013 (Additional file 1: Table S1). The proportion of patients in whom the PtCO_2_ increased from baseline by ≥4 mmHg at 26 min (6 min into the second oxygen-driven nebulisation) was also less than the proportion at 35 min (completion of the second oxygen-driven nebulisation), although this difference was not statistically significant: 10/45 (22%) and 14/45 (31%) respectively, paired difference in proportions (95% CI) 8.9% (− 3.3 to 20.9), *P* = 0.15.

The median (25th to 75th percentile) time taken for PtCO_2_ to return to baseline after cessation of the second nebulisation was 40 (40 to 45) minutes in the air group compared to 50 (45 to 50) minutes in the oxygen group, hazard ratio (95% CI) 1.59 (1.01 to 2.52), *P* = 0.047.

### PcapCO_2_ and pH

Data summaries for capillary blood gas sampling are shown in Table 3. The difference (95% CI) between oxygen and air for PcapCO_2_ after 35 min was 2.0 mmHg (1.1 to 2.8), *p* < 0.001. Thirteen (31.7%) participants receiving oxygen had a rise in PcapCO_2_ of ≥4 mmHg compared with three (7.7%) receiving air; risk difference (95% CI) 24% (7.5 to 40.5), *p* = 0.01. In addition to the two participants in whom the PtCO_2_ increased by ≥8 mmHg, there were two additional participants with capillary data receiving oxygen who had a rise in PcapCO_2_ of ≥8 mmHg and none from the air group. The mean (95% CI) difference in pH after 35 min was 0.015 units (0.008 to 0.024, p < 0.001) lower for oxygen nebulisation compared to air. One participant experienced a reduction in pH of 0.06 units (from 7.38 to 7.32) in association with a rise in PcapCO_2_ of 9 mmHg (55 to 64 mmHg).

**Table 3:** Capillary blood gas measurements according to randomised treatment

Time (mins)	P_cap_CO_2_ Mean (SD)		Difference^a^ (95% CI)	*P*
	Oxygen	Air		
0	40·2 (7·0) *N* = 43	38·5 (5·9) *N* = 41	–	–
35	42·6 (8·3) *N* = 41	39·0 (6·4) *N* = 39	2·0 (1.1 to 2·8)	< 0·001
Time (mins)	pH Mean (SD)		Difference^b^ (95% CI)	*P*
	Oxygen	Air		
0	7·42 (0·04) *N* = 43	7·44 (0·03) *N* = 41	–	–
35	7·41 (0·04) *N* = 41	7·43 (0·04) *N* = 39	-0·015 (− 0·024 to − 0·008)	< 0·001

*P*_*cap*_*CO*_*2*_ Capillary partial pressure of carbon dioxide

^a^P_cap_CO_2_ at 35 min, adjusted for baseline

^b^pH at 35 min, adjusted for baseline

### SpO_2_ and heart rate

**Fig. 3:**
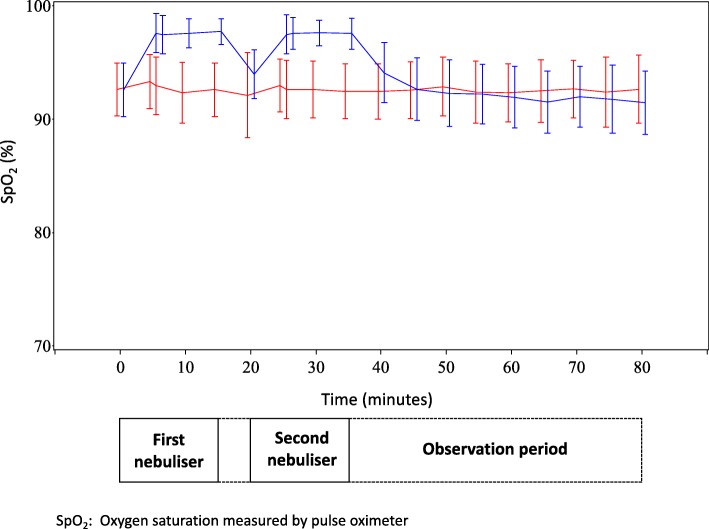
Time-course of SpO_2_ throughout study period (Blue = Oxygen-driven nebuliser group, Red = Air-driven nebuliser group)

The SpO_2_ was higher throughout both the nebulisation and initial washout periods in the oxygen compared with the air group (see Additional file 3: Table S2). Figure 3 shows the trend for the SpO_2_ in the oxygen group to fall below that of the air group after cessation of the second nebulisation. At the end of the observation period (80 min), the SpO_2_ was lower in the oxygen group (difference − 1.22%, 95% CI -2.04 to − 0.39, *p* = 0.004). The maximum reduction in SpO_2_ from baseline was 0·8% (95% CI -0.2 to 1.7, *P* = 0.10) lower after oxygen compared with air nebulisation. The heart rate was slower in the oxygen group at 35 min by 3.3 bpm (95% CI 0.31 to 6.25), *p* = 0.031 (see Additional file 1: Table S3).

### Methods of PCO_2_ measurement

Due to the requirement to change the primary outcome measure, a post-hoc analysis was undertaken to compare the two methods of measuring PaCO_2_. Based on data for 80 paired PtCO_2_ and PcapCO_2_ measurements at baseline and 35 min, the mean (SD) change in PtCO_2_ was 1.7 mmHg (2.2) with a range of − 2.5 to 8.0 mmHg, and the mean (SD) change in PcapCO_2_ was 1.7 mmHg (2.3), with a range − 3.0 to 9.0 mmHg. The estimate of bias for change in PcapCO_2_ minus PtCO_2_ was − 0.03 mmHg (95% CI -0.44 to 0.38), *P* = 0.89. The limits of agreement between PtCO_2_ and PcapCO_2_ were +/− 3.8 mmHg for each individual measurement obtained.

## Discussion

In this study, oxygen-driven nebulisation increased the PtCO_2_ in hospital in-patients with an AECOPD compared with air-driven nebulisation. Despite the small mean increase in PtCO_2_ of 3.4 mmHg, the physiological relevance of this response is suggested by the increase in PtCO_2_ of at least 4 mmHg in 18/45 (40%) of participants receiving oxygen-driven nebulisation, whereas no patient had an increase of 4 mmHg or more following air-driven nebulisation. The clinical relevance of this physiological response is suggested by the requirement to withdraw one participant during the second oxygen-driven nebulisation due to the PtCO_2_ increasing by > 10 mmHg, and the increase of PtCO_2_ or PcapCO_2_ of at least 8 mmHg in 4/45 (9%) patients receiving oxygen-driven nebulisation, one of whom had a fall in pH of 0.06 into the acidotic range (7.32). These findings suggest that air-driven nebulised bronchodilator therapy represents an important component of the conservative titrated oxygen regimen which has been shown to reduce the risk of hypercapnia, acidosis and mortality in AECOPD [1].

There are a number of methodological issues relevant to the interpretation of the study findings. Both the randomised controlled design and double-blinding of this study allow for robust and reliable data capture. The length of the nebuliser regimen was chosen to ensure adequate time for complete nebulisation to occur, and to replicate ‘real-world’ back to back treatments in the acute setting, by using two nebulisations separated by five minutes. It is possible that the magnitude of the differences in PCO_2_ and pH may be even larger with continuous nebulisation which may occur in patients with severe exacerbations not responding to initial treatment or if the nebuliser is inadvertently left in place. The safety-based exclusion criteria of a baseline PtCO_2_ > 60 mmHg and an oxygen requirement of ≥4 L/minute (to maintain target SpO_2_ of 88 to 92%), effectively excluded patients with the most severe exacerbations of COPD.

Whilst respiratory rate and neurological symptoms were not formally assessed as outcome measures, no adverse events were identified during the interventions. However, we acknowledge that if changes in PCO_2_ and pH of this magnitude occurred in more severe patients at the time of their presentation, they would have been at risk of symptoms of hypercapnia and respiratory acidosis, and the requirement to escalate treatment.

The original primary outcome measure and time of measurement was PcapCO_2_ after 35 min. Following the first 14 study participants, it was evident that obtaining adequate amounts of blood to fill the capillary tubes from some participants was difficult or impossible to the extent that 4 out of 14 participants had one or more missed samples. For this reason, the primary outcome was changed to PtCO_2_ after 35 min. In other words, the method of capturing the change in PCO_2_ was revised, rather than the outcome itself. PtCO_2_ monitoring enabled continuous assessment to be undertaken, and is accurate in AECOPD, [9] and other acute settings [10–12]. The validity of this method was confirmed by the post hoc analysis of 80-paired samples, where each capillary blood gas sample obtained had a corresponding PtCO_2_ measurement at the same time-point. This showed that the difference between the PcapCO_2_ and PtCO_2_ in the mean change from baseline was − 0·03 mmHg with 95% confidence intervals of − 0.44 to 0.38 mmHg. This data suggests that the use of PtCO_2_ measurements did not adversely affect our ability to determine change in PcapCO_2_ from baseline.

We did not investigate the potential mechanisms by which oxygen driven nebulisation increases PtCO_2_. However as demonstrated in mechanistic studies of oxygen therapy in COPD, it is likely to be due to the combination of a reduction in respiratory drive, release of hypoxic pulmonary vasoconstriction, absorption atelectasis, and the Haldane effect [13, 14]. Furthermore, the study was not designed to assess costs related to each regimen, however it is reasonable to assume that improved clinical outcomes seen by avoiding a rise in PtCO_2_ and associated acidosis, would lead to a reduction in healthcare costs.

The findings from our study complement those of our previous randomised controlled trial of a similar design in stable COPD patients in the clinic setting, in which there was a mean PtCO_2_ difference between the oxygen- and air-driven nebulisation treatment arms of 3.1 mmHg (95% CI 1·6 to 4·5), *p* < 0·001, after 35 min. [3] In that study one of the 24 subjects was withdrawn due to an increase in PtCO_2_ of 10 mmHg after 15 min of the first oxygen-driven nebulisation. As with the previous study, an increase in PtCO_2_ occurred within 5 min, indicating the rapid time course of this physiological response. We had anticipated a greater effect in this current study as the patients had acute rather than stable COPD however the magnitude of the effect was similar, probably reflecting the similar severity of airflow obstruction, with a mean predicted FEV_1_ of 35% and 27% in this and the previous study respectively.

The two previous open crossover studies of inpatients with AECOPD both showed oxygen-driven nebulisation worsened hypercapnia in patients with Type 2 respiratory failure [4, 5]. Gunawardena et al. [4] studied 16 patients with COPD and reported that only those with carbon dioxide retention at baseline (*n* = 9) demonstrated a rise in PaCO_2_ after 15 min (mean of 7·7 mmHg), and one patient had a rise of 22 mmHg. Similarly, O’Donnell et al [5] reported that 6/10 patients, all with carbon dioxide retention at baseline, showed a rise in PaCO_2_ after 10 min (mean of 12.5 mmHg).

The current BTS guidelines recommend air-driven nebulisation and, if this is not available in the ambulance service, the maximum use of 6 min for an oxygen-driven nebuliser. This is based on the rationale that most of the nebulised medication will have been delivered, and is categorised as grade D evidence [6]. We observed the mean time for dissipation of salbutamol solution from the nebuliser chamber of 5.2 min confirming that 6 min is adequate for salbutamol delivery. The proportion of participants with a PtCO_2_ increase ≥4 mmHg was lower after 6 min than 15 min, suggesting some amelioration of risk with the shorter nebulisation treatment. Alternative methods of bronchodilator delivery include air-driven nebulisers or multiple metered dose inhaler actuations via a spacer [15].

The potential for rebound hypoxia after abrupt cessation of oxygen therapy has been observed both in the treatment of asthma and COPD [9, 16, 17]. We identified some evidence consistent with this phenomenon which is a potentially important yet poorly recognised clinical issue.

## Conclusions

In summary, air-driven nebulisation avoids the potential risk of increasing the PaCO_2_ associated with oxygen-driven bronchodilator administration in AECOPD. We propose that air-driven bronchodilator nebulisation is preferable to oxygen-driven nebulisation in AECOPD, and that when the use of oxygen-driven nebulisation is unavoidable, PtCO_2_ is monitored if possible.

## Additional files

Additional file 1: Original Protocol. (DOC 209 kb)

Additional file 2: Protocol Version 2.0. (PDF 157 kb)

Additional file 3: Online supplement - **Table S1.** PtCO_2_ change ≥4 mmHg according to randomised treatment. **Table S2.** SpO_2_ mixed linear model comparisons: Oxygen minus Air. **Table S3.** Heart Rate mixed linear model comparisons: Oxygen minus Air. (DOC 80 kb)

## Abbreviations

AECOPD: Acute exacerbation of chronic obstructive pulmonary disease
BTS: British thoracic society
COPD: Chronic obstructive pulmonary disease
FEV_1_: Forced expiratory volume over 1 s
FVC: Forced vital capacity
MRINZ: Medical research institute of new zealand
PaCO_2_: Partial pressure of arterial carbon dioxide
PcapCO_2_: Partial pressure of capillary carbon dioxide
PIS: Participant information sheet
PtCO_2_: Partial pressure of transcutaneous carbon dioxide
SD: Standard deviation
Sentec: Transcutaneous monitor brand
SpO_2_: Oxygen saturation measured by oximetry
StO_2_: Oxygen saturation measured by transcutaneous monitor
