# Supplementary material for: Oxygen versus air-driven nebulisers for exacerbations of chronic obstructive pulmonary disease: a randomised controlled trial
Source: BMC Pulm Med. 2018 Oct 3;18:157. doi: 10.1186/s12890-018-0720-7 (PMC6171193; doi:10.1186/s12890-018-0720-7)
Supplement: Supplementary file 1 — Original Protocol. (DOC 209 kb) [file 12890_2018_720_MOESM1_ESM.doc]

##

Protocol

**Oxygen versus air driven nebulisers in exacerbations of COPD.**

**Full title:**

Randomised double blind study to investigate the effect of oxygen versus air driven nebulisers on partial pressure of arterial carbon dioxide in patients with an exacerbation of chronic obstructive pulmonary disease.

**Protocol No. 1**

Universal Trial Number: U111111634438

ANZCTR Number: <<to insert>>

HDEC Approval Number: <<to insert>>

Funding: *HRC partnership grant in conjunction with MRINZ*

**Investigators:**

Geroge Bardsley

Michael Richards

Pip Shirtcliffe

Janine Pilcher

Mark Holliday

Mark Weatherall

Richard Beasley

**Contact:**

Dr Janine Pilcher

Medical Research Institute of New Zealand

Private Bag 7902, Wellington, New Zealand, Telephone: +64-4-805 0241

Facsimile: +64-4-472 9224, Email: [janine.pilcher@mrinz.ac.nz](mailto:janine.pilcher@mrinz.ac.nz)

# Background

Acute exacerbations of chronic obstructive pulmonary disease (COPD) result in over 9,000 hospital admissions every year in New Zealand (NZ).^1^ Nebulised medication is routinely administered to patients with an acute exacerbation and can be delivered via either high flow oxygen or air.

Oxygen driven nebulisers expose patients to high concentrations of inspired oxygen. The risks of high concentration oxygen have been shown by the recent randomised controlled trial (RCT) in which high concentration oxygen therapy (supplementary oxygen 8-10 L/min and high flow oxygen-driven nebulisers for bronchodilator delivery) caused a 2.4-fold increased risk of death compared with titrated oxygen therapy (supplementary oxygen titrated as needed to achieve an oxygen saturation of 88-92% and air-driven nebulisers for bronchodilator delivery) in the management of severe exacerbations of COPD.^2^ Hypercapnia, an elevated arterial carbon dioxide tension (PaCO_2_), is a known risk of high concentration oxygen therapy in patients with COPD,^3 4^ and oxygen driven nebuliser use has been associated with marked increases in PaCO_2_, which resulted in stupor, seizures and death.^5-7^

The British Thoracic Society (BTS) recommends titrated oxygen therapy to achieve oxygen saturations of 88-92% and air-driven nebuliser use for acute exacerbations of COPD.^8^ It is recommended that if air-driven nebulisers are unavailable, use of oxygen driven nebulisers should be limited for up to six minutes.^8^ We have shown that air-driven nebulisers will prevent the increase in PaCO_2_ that results from use of oxygen-driven nebulisers in patients with stable COPD.^9^ However, there are only two published RCTs in patients with acute exacerbations of COPD.^10 11^ While acknowledging the important limitations of these studies, including lack of blinding, administration of single bronchodilator doses and low power, they did identify increased risk of an elevation in PaCO_2_ in COPD patients with hypercapnia.^10 11^

The risks of oxygen delivered nebulisation in acute severe exacerbations of COPD need to be robustly defined to identify whether the widespread implementation of air driven nebulisers is required to ensure the safe delivery of bronchodilators by nebulisation to this at-risk patient group.

# Design & Objectives

## Design

A parallel group double blinded RCT comparing the effect of air versus oxygen driven bronchodilator nebulisation on PaCO_2_, pH and oxygen saturation (SpO_2_) in patients admitted to hospital with an acute exacerbation of COPD. Ninety patients will be recruited and randomised to receive two 15 minute administrations of salbutamol by nebulisation, delivered by air or oxygen at a flow rate of 8L/min.

## Objectives

1. To compare the effects of air versus oxygen driven bronchodilator nebulisation on PaCO_2_, pH and SpO_2_ in acute severe exacerbations of COPD
2. To determine if the BTS recommendation of six minute oxygen driven nebuliser use avoids the risk of an oxygen induced increase in PaCO_2_

## Hypotheses

1. Oxygen driven bronchodilator nebulisation results in increases in PaCO_2_ in severe exacerbations of COPD compared with air driven bronchodilator nebulisation
2. Oxygen driven bronchodilator nebulisation results in a reduction in pH in severe exacerbations of COPD compared with air-driven bronchodilator nebulisation
3. The increase in PaCO_2_ and reduction in pH secondary to oxygen-driven nebuliser use are clinically significant in some patients, defined as >8mmHg change in PaCO_2_ and >0.1 change in pH.
4. Clinically significant elevations in PaCO_2_ secondary to oxygen-driven nebuliser use may occur within six minutes^11^ of commencement of nebulisation in some patients
5. Cessation of oxygen-driven bronchodilator nebulisation may result in rebound hypoxaemia

# Outcome variables

## Comparator groups

Outcome variables will be compared between the air versus oxygen driven nebuliser regimens.

## outcOme measurements

Arteriolised earlobe blood gas samples (AeBG) will measure PaCO_2_ (P_art_CO_2_) and pH. Which ear the sample is taken from will be randomised. The sample will be collected in capillary tubes and analysed at the Wellington Regional Hospital or Hutt Valley Hospital laboratory. The TOSCA 500 (Radiometer, Basle, Switzerland) will be placed on the contra lateral ear to the one the AeBG was taken to measure transcutaneous PaCO_2_ (PtCO_2_), oxygen saturation (StO_2_) and heart rate. Finger pulse oximetry will measure oxygen saturation (SpO_2_).

## Primary outcome variable

1. P_art_CO_2_ at completion of the second nebulisation (immediately prior to t=35min), adjusted for baseline.

## Secondary outcome variables

1. pH at completion of the second nebulisation (immediately prior to t=35 min), adjusted for baseline.
2. PtCO_2_ at six minutes after the initiation of the first and second nebulisers (t=6 min and t=26 min)
3. PtCO_2_, SpO_2_ and heart rate at 5 minute intervals from t=0 to t=80 min
4. Proportion of participants with >4mmHg (physiologically significant)^12 13^ and >8mmHg (clinically significant)^12 13^ increase in P_art_CO_2_ or PtCO_2_ from baseline
5. Number of intervention terminations due to a rise in PtCO_2_ >10mmHg
6. The proportion of patients with a reduction in pH of < 0.1 from baseline
7. Proportion of patients who required initiation an increase in the flow of oxygen therapy during the regimen and observation periods.

# Study Subjects

## Inclusion criteria

1. Ninety patients admitted to Hutt Valley and Wellington Regional Hospital medical inpatient wards with a primary admission diagnosis of an exacerbation of COPD.

## Exclusion criteria

1. Age <40 years at time of randomisation
2. Requirement for assisted non-invasive ventilation at time of randomisation
3. Baseline PtCO_2_ >60mmHg at time of randomisation
4. Requirement for >4L/min of oxygen via nasal cannulae to maintain SpO_2_ between 88-92% prior to or during titration
5. Any other condition which, at the investigator’s discretion, is believed may present a safety risk or impact the feasibility of the study or the study results.

# Randomisation and blinding

## Randomisation

Randomisation will be 1:1 via a block randomised (block size 6) computer generated sequence, provided by the study statistician independent of recruitment and assessment of participants. The allocated intervention will be stored in an opaque sealed envelope and opened at the time of randomisation by the un-blinded investigator.

## Blinding

### Blinding of the blinded investigator during the study visit

The blinded investigator will be sat behind a portable screen so that they cannot see the participant or pulse oximeter screen. The participant will be asked not to comment out loud whether they are wearing their mask or nasal prongs.

### Blinding of the laboratory technician

The laboratory technician analysing the AeBG sample will be masked to the randomised treatment.

### Blinding of the participant

The oxygen and air cylinders will not be distinguishable from each other to maintain blinding of the participant as to which gas their nebuliser is driven by. Participants will not be advised which treatment regimen they are randomised to or of the detail that one regimen involves removal of nasal prongs (oxygen-driven) and the other does not (air driven). They will be informed, however, that their oxygen saturations will be monitored closely and if there are any concerns the regarding low oxygen saturation the un-blinded investigator will provide appropriate oxygen therapy immediately.

### Roles of the un-blinded investigator

1. AeBG sampling
2. Randomisation
3. Administration of the randomised intervention
4. Recording and monitoring of finger probe SpO_2_
5. Increase oxygen therapy if SpO_2_ <85%.

### Roles of the blinded investigator

1. Recording of PtCO_2_
2. Recording of heart rate
3. Advise the cessation of the intervention should the PtCO_2_ rise by >10mmHg from baseline.

# Nebuliser equipment

1. Hudson RCI Micro Mist Nebuliser Masks (Hudson RCI, Durham, North Carolina, USA) will be used to deliver the air and oxygen driven nebulised bronchodilator.
2. Salbutamol 2.5 mg will be used for each 15 minute nebulisation
3. Oxygen and air to drive the nebulisation will be supplied in compressed portable cylinders (size D).

# Study visit conduct

## Recruitment, TIMING and consent

Potentially eligible participants will be identified on the wards and invited to take part in the study. Patients can be recruited at any time during their medical admission; however the study visit should be timed to deliver the nebuliser regimen as close as possible to the time of the next prescribed bronchodilator dose. Written Informed consent will take place prior to any study specific procedures.

## Demographic data collection

### The following will be collected on medical history

1. Primary reason for admission
2. Age and ethnicity
3. Pack year history
4. History of:
   1. Long term oral prednisone use
   2. Home oxygen use
   3. Home nebuliser use
   4. Previous hypercapnic respiratory failure
   5. Previous assisted ventilation
5. Medical comorbidities
6. Date, time, dose and method (nebuliser, spacer or inhaler only) of last bronchodilator medication(s)
7. Date, time, dose and method (nebuliser, spacer or inhaler only) of the next prescribed bronchodilator medication(s)

### The following will be measured

1. Weight and height

NOTE: Should the participant be too unwell to take these measures, data may be collected from clinical records or patient history. The method of collecting this data (investigator measurement, clinical records or patient history must be documented).

1. In participants that are able to perform spirometry, this will be done using a using a Micro Spirometer (Micro Medical Ltd, Rochester, UK) to measure FEV_1_ and FVC, and according to ATS/ ERS criteria.^14 15^

## STUDY SET UP

### TOSCA Calibration

The TOSCA screen displaying StO_2_ is to be covered. The TOSCA will be attached to the participant’s earlobe as per manufacturer’s instructions. A calibration period of at least 10 minutes will take place. This will be followed by measurements of PtCO_2_ every 2 minutes until consecutive measurements are the same. The TOSCA temperature must display 42 degrees before continuing.

### Pulse oximeter application

The finger oximeter will then be placed on the participant’s index finger. The StO_2_ screen will be uncovered on the TOSCA and the un-blinded investigator will record the time, StO2 and SpO_2_. The TOSCA screen displaying StO_2_ will be covered again and until t=80.

### Blinded investigator set up

From this point on and until t=80 minutes the blinded investigator and TOSCA unit will go behind a screen to avoid visualisation of the participant and pulse oximeter screen.

## Wash in period

### Baseline measures

1. The un-blinded investigator will record the time, SpO_2_ and nasal cunnulae oxygen therapy flow (if any)
2. The blinded investigator will record the time, PtCO_2_ and heart rate

### Intervention

For at least 15 minutes the un-blinded investigator will titrate oxygen therapy via nasal cannulae to achieve an SpO_2_ of 88-92%. Note this may mean the participant breathes room air. If the participant requires > 4L/min via nasal cannulae, they will be excluded.

### Monitoring and measurements by the un-blinded investigator

1. Every 5 minutes record the time and SpO_2_.
2. Monitor the SpO_2_ and if the nasal cannulae oxygen flow is changed, document the time and new oxygen flow

### Monitoring and measurements by the blinded investigator

1. Every 5 minutes record the time, PtCO_2_ and heart rate.

## Randomised regimen

### Baseline measures

1. At the end of the washout and immediately prior to the randomised regimen the un-blinded investigator will take an AeBG, as per the MRINZ SOP. On visualisation of blood entering the capillary tube they will ask the blinded investigator to record the time and PtCO_2_.
2. Immediately after the AeBG sampling, the following baseline (t=0 min) measurements will be made:
   - 1. The un-blinded investigator will record the time, SpO_2_ and nasal cannulae oxygen therapy flow (if any)
     2. The blinded investigator will record the time, PtCO_2_ and heart rate

### Randomisation

The randomisation envelope will be opened by the un-blinded investigator and regimens applied as below.

### Regimens

Air driven nebuliser regimen

1. t=0-15 min: 2.5 mg salbutamol by nebulisation, delivered by air at a flow rate of 8L/min
2. t=15-20 min: Removal of nebuliser mask
3. t=20-35 min: 2.5 mg salbutamol by nebulisation, delivered by air at a flow rate of 8L/min.

The nebuliser is to continue running for the entire 15 minutes at t=0-15 min and t=20-35 min, even if the bronchodilator medication has dissipated.

Oxygen therapy is to be continued to be titrated by the un-blinded investigator using nasal cannulae worn under the nebuliser mask.

Oxygen driven nebuliser regimen

1. t=0-15 min: 2.5 mg salbutamol by nebulisation, delivered by oxygen at a flow rate of 8L/min
2. t=15-20 min: Removal of nebuliser mask
3. t=20-35 min: 2.5 mg salbutamol by nebulisation, delivered by oxygen at a flow rate of 8L/min.

The nebuliser is to continue running for the entire 15 minutes at t=0-15 min and t=20-35 min, even if the bronchodilator medication has dissipated.

Any nasal cannulae oxygen therapy is to be discontinued and nasal prongs removed, during t=0-15 min and t=20-35 min. Oxygen must be restarted at the same flow given prior to nebuliser delivery (i.e. at t=15 the oxygen must be started at the flow delivered at t=0, and at t=35 it must be started again at the flow delivered at t=20 minutes).

### Monitoring and measurements by the un-blinded investigator

1. At t= 5, 6, 10, 15, 20, 25, 26, 30, 35 record the time and SpO_2_.
2. Monitor the SpO_2_ and if the nasal cannulae oxygen flow is changed, document the time and new oxygen flow (air driven regimen only)
3. Document the time the nebulised medication canister becomes empty.
4. As close as possible prior to the completion of the second nebuliser at t=35 minutes, an AeBG measurement will be taken, as per the MRINZ SOP. On visualisation of blood entering the capillary tube un-blinded investigator ask blinded investigator to record the time and PtCO_2_.

NOTE: The t=20 measure is to occur immediately prior to initiation of the 2^nd^ nebuliser and the t= 15 and t=35 measures are to occur immediately prior to cessation of nebuliser delivery

### Monitoring and measurements by the blinded investigator

1. At t= 5, 6, 10, 15, 20, 25, 26, 30, 35 record the time, PtCO_2_ and heart rate
2. Continuously monitor PtCO_2_, and should it increase by >10mmHg from baseline measure (t=0), indicate this to the un-blinded investigator so they can terminate the regimen intervention
3. Record the time and PtCO_2_ during arterialised earlobe blood gas sampling, as prompted by the un-blinded investigator.

NOTE: The t=20 measure is to occur immediately prior to initiation of the 2^nd^ nebuliser and the t= 15 and t=35 measures are to occur immediately prior to cessation of nebuliser delivery

## Post Regimen monitoring

### Intervention

The un-blinded investigator is to document the oxygen flow instituted at the end of the second nebuliser as Flow A. Until t=80 oxygen will be delivered at Flow A via nasal cannulae except if the SpO_2_ falls to <85%. In this case the flow is to be titrated until the 88-92% target saturation range is met.

### Measurements and monitoring by the un-blinded investigator

1. At t= 40, 45, 50, 55, 60, 65, 70, 75, 80 record the time and SpO_2_
2. Monitor the SpO_2_ and if the nasal cannulae oxygen flow is changed, document the time and new oxygen flow

### Measurements and monitoring by the blinded investigator

1. At t= 40, 45, 50, 55, 60, 65, 70, 75, 80 record the time, PtCO_2_ and heart rate.

## Study schema

|  | Titration of oxygen to SpO_2_ of 90%* | Delivery of oxygen therapy at Flow A* | | | |
| --- | --- | --- | --- | --- | --- |
|  | |  | | | |
| Recruitment and consent  Wash in period | | Air-driven nebuliser administration |  | Air-driven nebuliser administration | Observation period |
|  |  |  |  |  |  |
|  |  | Oxygen-driven nebuliser administration^#^ |  | Oxygen-driven nebuliser administration^#^ | Observation period |
|  | |  | | | |
| Regimen baseline measures** &Randomisation  Wash-in baseline measures** | |  | | | |
| Time  >-15^##^  (min) | 0 | 15 | 20 | 35 | 80 |
| Measured/  recorded by  un-blinded investigator: | P_art_CO_2_ & pH  P_art_CO_2_ & pH | | | | |
|  | SpO_2_  Baselines, every 5 minutes  & at t=6 and t=26 min  Any SpO_2_ <85% and alteration to nasal cannulae oxygen flow*  Time at which nebulised solution dissipated | | | | |
| Measured/  recorded by blinded  investigator | PtCO_2_ & HR  Baselines, every 5 minutes  & at t=6 and t=26 min | | | | |

*From Time 0 only.

^#^Nasal cannulae oxygen delivery will be stopped during this time.

** Baseline measures are: Time; SpO_2_; oxygen flow via nasal cannulae, if any (Flow A); PtCO_2_ and HR

^##^ Minimum time period of 15 minutes prior to arteriolised earlobe blood gas

HR: Heart rate, P_art_CO_2_: Arterialised partial pressure of carbon dioxide, PtCO_2_: Transcutaneous partial pressure of carbon dioxide, SpO_2_: oxygen saturations

# Methodology notes

## Wash in, regimen and observation periods

Delivery of two 15 minute nebulisers with a 5 minute gap with a 2.5mg salbutamol dose were selected to represent real-life nebuliser delivery. Titration of oxygen before and during the regimens represents recommended evidence based on best practice.^2 8^

Maintenance of any nasal cannulae oxygen therapy at Flow A during the observation period is designed to assess the risk of rebound hypoxia (a reduction in SpO_2_ following abrupt cessation of oxygen therapy to below the baseline level prior to instituting oxygen therapy). To detect any rebound hypoxemia a constant fraction of inspired oxygen is required. The results would represent the risk of rebound hypoxia in the situation that oxygen saturation monitoring does not occur following the abrupt cessation of high concentration.

## Primary outcome

Elevated PaCO_2_, is potentially harmful physiological response to oxygen administration^2 3 8-11 16-20^ and predictor of adverse clinical outcome.^21 22^ This allows a robust power calculation^9^ and direct comparison to previous case and interventional studies that have investigated the use of oxygen driven nebulisers.^9-11^

## CLINICALLY SIGNIFICANT outcomes

A rise in PtCO_2_ from baseline of >4mmHg is considered a physiologically significant change and >8mmHg a clinically significant change, based on previous definitions.^12^ A change in pH >0.1 is based on the magnitude of the difference in pH that is expected to result from an increase in PaCO_2_ and >8mmHg and has clinical relevance in as a marker of requirement for NIV in an exacerbation of COPD.^23^

## Arteriolised earlobe blood gas sampling

AeBG allows accurate measurement of P_art_CO_2_^24^ and pH,^24 25^ and is a less invasive alternative to ABG measurement. The earlobe is the preferred site.^24^ pH is a valuable outcome measure as it is an independent predictor of death in exacerbations of COPD.^26-28^

## TOSCA monitoring

The TOSCA provides continuous and non-invasive PtCO_2_ monitoring. The accuracy of PtCO2 monitoring has been shown in a variety of settings including in healthy subjects,^29^ acute exacerbation of COPD,^30^ sleep disorders,^31^ critical illness,^32^ and other patients.^33 34^

# Safety Monitoring

There will be a Data Monitoring Safety Board comprising independent physicians, which will review data from any participants in which an SAE or rise in PtCO_2_ of >10mmHg occurred. This will include experts independent from the study.

# Power and Statistical Methods

A difference in PaCO_2_ of 4 mmHg represents a physiologically significant change.^12 13^ In our controlled study of oxygen versus air driven nebulisers in stable COPD the standard deviation of PtCO_2_ was 5.5.^9^ With 90% power and alpha of 5% this requires a total of 82 patients to detect a 4 mmHg difference. We anticipate a drop-out rate of <10% so our target recruitment is 90 patients. Analysis will be by intention to treat. Our primary analysis is ANCOVA with P_art_CO_2_ as the response variable, and randomised treatment and baseline P_art_CO_2_ as co-variates. For other continuous outcome variables we will also use similar ANCOVA. Exploratory analyses for PtCO_2_, heart rate and SpO_2_ taken at five minute intervals will be presented graphically and analysed by mixed linear models.

# Note on changes to protocol since previous version

This is Version 1, which was submitted in the initial application of ethics approval. There are no previous versions for comparison.

# References

1. Martin P, Glasgow H, Patterson J. Chronic obstructive pulmonary disease (COPD): smoking remains the most important cause. *N Z Med J* 2005;118(1213):U1409.

2. Austin MA, Wills KE, Blizzard L, Walters EH, Wood-Baker R. Effect of high flow oxygen on mortality in chronic obstructive pulmonary disease patients in prehospital setting: randomised controlled trial. *BMJ* 2010;341:c5462.

3. Murphy R, Driscoll P, O'Driscoll R. Emergency oxygen therapy for the COPD patient. *Emerg Med J* 2001;18(5):333-9.

4. New A. Oxygen: kill or cure? Prehospital hyperoxia in the COPD patient. *Emerg Med J* 2006;23(2):144-6.

5. Austin SJ, Chan C. Oxygen as a driving gas for nebulisers: safe or dangerous? *Br Med J (Clin Res Ed)* 1984;288(6415):488.

6. Lim TK, Tan WC. Acute carbon dioxide narcosis during inhalational therapy with oxygen powered nebulizers in patients with chronic airflow limitation. *Ann Acad Med Singapore* 1985;14(3):439-41.

7. Hadfield JW, Stinchcombe SJ, Bateman JR. Oxygen as a driving gas for nebulisers: safe or dangerous? *Br Med J (Clin Res Ed)* 1984;288(6419):795.

8. O'Driscoll BR, Howard LS, Davison AG. BTS guideline for emergency oxygen use in adult patients. *Thorax* 2008;63 Suppl 6:vi1-68.

9. Edwards L, Perrin K, Williams M, Weatherall M, Beasley R. Randomised controlled crossover trial of the effect on PtCO2 of oxygen-driven versus air-driven nebulisers in severe chronic obstructive pulmonary disease. *Emerg Med J* 2012;29:894-898

10. Gunawardena KA, Patel B, Campbell IA, MacDonald JB, Smith AP. Oxygen as a driving gas for nebulisers: safe or dangerous? *Br Med J (Clin Res Ed)* 1984;288(6413):272-4.

11. O'Donnell D, Kelly CP, Cotter P, Clancy L. Use of oxygen driven nebulizer delivery systems for beta-2 agonists in chronic bronchitis. *Ir J Med Sci* 1985;154(5):198-200.

12. Wijesinghe M, Williams M, Perrin K, Weatherall M, Beasley R. The effect of supplemental oxygen on hypercapnia in subjects with obesity-associated hypoventilation: a randomized, crossover, clinical study. *Chest* 2011;139(5):1018-24.

13. Perrin K, Wijesinghe M, Healy B, Wadsworth K, Bowditch R, Bibby S, et al. Randomised controlled trial of high concentration versus titrated oxygen therapy in severe exacerbations of asthma. *Thorax* 2011;66(11):937-41.

14. Miller MR, Hankinson J, Brusasco V, Burgos F, Casaburi R, Coates A, et al. Standardisation of spirometry. *Eur Respir J* 2005;26(2):319-38.

15. Miller MR, Crapo R, Hankinson J, Brusasco V, Burgos F, Casaburi R, et al. General considerations for lung function testing. *Eur Respir J* 2005;26(1):153-61.

16. Prime FJ, Westlake EK. The respiratory response to CO2 in emphysema. *Clin Sci (Lond)* 1954;13(3):321-32.

17. Westlake EK, Simpson T, Kaye M. Carbon dioxide narcosis in emphysema. *Q J Med* 1955;24(94):155-73.

18. Campbell EJ. A method of controlled oxygen administration which reduces the risk of carbon-dioxide retention. *Lancet* 1960;2(7140):12-4.

19. Massaro DJ, Katz S, Luchsinger PC. Effect of various modes of oxygen administration on the arterial gas values in patients with respiratory acidosis. *Br Med J* 1962;2(5305):627-9.

20. Warrell DA, Edwards RH, Godfrey S, Jones NL. Effect of controlled oxygen therapy on arterial blood gases in acute respiratory failure. *Br Med J* 1970;1(5707):452-5.

21. Groenewegen KH, Schols AM, Wouters EF. Mortality and mortality-related factors after hospitalization for acute exacerbation of COPD. *Chest* 2003;124(2):459-67.

22. Soler-Cataluna JJ, Martinez-Garcia MA, Roman Sanchez P, Salcedo E, Navarro M, Ochando R. Severe acute exacerbations and mortality in patients with chronic obstructive pulmonary disease. *Thorax* 2005;60(11):925-31.

23. NICE. Chronic obstructive pulmonary disease. National clinical guideline on management of chronic obstructive pulmonary disease in adults in primary and secondary care. *Thorax* 2004;59 Suppl 1:1-232.

24. Zavorsky GS, Cao J, Mayo NE, Gabbay R, Murias JM. Arterial versus capillary blood gases: a meta-analysis. *Respir Physiol Neurobiol* 2007;155(3):268-79.

25. Murphy R, Thethy S, Raby S, Beckley J, Terrace J, Fiddler C, et al. Capillary blood gases in acute exacerbations of COPD. *Respir Med* 2006;100(4):682-6.

26. Denniston AK, O'Brien C, Stableforth D. The use of oxygen in acute exacerbations of chronic obstructive pulmonary disease: a prospective audit of pre-hospital and hospital emergency management. *Clin Med* 2002;2(5):449-51.

27. Roberts CM, Stone RA, Buckingham RJ, Pursey NA, Lowe D. Acidosis, non-invasive ventilation and mortality in hospitalised COPD exacerbations. *Thorax* 2011;66(1):43-8.

28. Plant PK, Owen JL, Elliott MW. One year period prevalence study of respiratory acidosis in acute exacerbations of COPD: implications for the provision of non-invasive ventilation and oxygen administration. *Thorax* 2000;55(7):550-4.

29. Fuke S, Miyamoto K, Ohira H, Ohira M, Odajima N, Nishimura M. Evaluation of transcutaneous CO2 responses following acute changes in PaCO2 in healthy subjects. *Respirology* 2009;14(3):436-42.

30. Cox M, Kemp R, Anwar S, Athey V, Aung T, Moloney ED. Non-invasive monitoring of CO2 levels in patients using NIV for AECOPD. *Thorax* 2006;61(4):363-4.

31. Senn O, Clarenbach CF, Kaplan V, Maggiorini M, Bloch KE. Monitoring carbon dioxide tension and arterial oxygen saturation by a single earlobe sensor in patients with critical illness or sleep apnea. *Chest* 2005;128(3):1291-6.

32. Rodriguez P, Lellouche F, Aboab J, Buisson CB, Brochard L. Transcutaneous arterial carbon dioxide pressure monitoring in critically ill adult patients. *Intensive Care Med* 2006;32(2):309-12.

33. McVicar J, Eager R. Validation study of a transcutaneous carbon dioxide monitor in patients in the emergency department. *Emerg Med J* 2009;26(5):344-6.

34. Perrin K, Wijesinghe M, Weatherall M, Beasley R. Assessing PaCO2 in acute respiratory disease: accuracy of a transcutaneous carbon dioxide device. *Intern Med J* 2011;41(8):630-3.
