# Supplementary material for: Oxygen versus air-driven nebulisers for exacerbations of chronic obstructive pulmonary disease: a randomised controlled trial
Source: BMC Pulm Med. 2018 Oct 3;18:157. doi: 10.1186/s12890-018-0720-7 (PMC6171193; doi:10.1186/s12890-018-0720-7)
Supplement: Supplementary file 2 — Protocol Version 2.0. (PDF 157 kb) [file 12890_2018_720_MOESM2_ESM.pdf]

Protocol

Oxygen versus air driven nebulisers in exacerbations of COPD.

Full title:

Randomised double blind study to investigate the effect of oxygen versus air driven nebulisers on partial pressure of arterial carbon dioxide in patients with an exacerbation of chronic obstructive pulmonary disease.

Short title:

Oxygen versus air driven nebulisers in COPD exacerbation

Protocol No. 2.0

Universal Trial Number: U111111634438

ANZCTR Number: 367653

HDEC Approval Number: 14/NTB/200

Funding: *HRC partnership grant in conjunction with MRINZ*

Investigators:

George Bardsley
Steve McKinstry
Pip Shirtcliffe
Janine Pilcher
Mark Holliday
Mark Weatherall
Richard Beasley

Contact:

Dr George Bardsley, Medical Research Institute of New Zealand

Private Bag 7902, Wellington, New Zealand, Telephone: +64-4-805 0241

Facsimile: +64-4-472 9224, Email: george.bardsley@mrinz.ac.nz

1. Background

Acute exacerbations of chronic obstructive pulmonary disease (COPD) result in over 9,000 hospital admissions every year in New Zealand (NZ).[1] Nebulised medication is routinely administered to patients with an acute exacerbation and can be delivered via either high flow oxygen or air.

Oxygen driven nebulisers expose patients to high concentrations of inspired oxygen. The risks of high concentration oxygen have been shown by the recent randomised controlled trial (RCT) in which high concentration oxygen therapy (supplementary oxygen 8-10 L/min and high flow oxygen-driven nebulisers for bronchodilator delivery) caused a 2.4-fold increased risk of death compared with titrated oxygen therapy (supplementary oxygen titrated as needed to achieve an oxygen saturation of 88-92% and air-driven nebulisers for bronchodilator delivery) in the management of severe exacerbations of COPD.[2] Hypercapnia, an elevated arterial carbon dioxide tension ($PaCO_2$), is a known risk of high concentration oxygen therapy in patients with COPD,[3 4] and oxygen driven nebuliser use has been associated with marked increases in $PaCO_2$, which resulted in stupor, seizures and death.[5-7]

The British Thoracic Society (BTS) recommends titrated oxygen therapy to achieve oxygen saturations of 88-92% and air-driven nebuliser use for acute exacerbations of COPD.[8] It is recommended that if air-driven nebulisers are unavailable, use of oxygen driven nebulisers should be limited for up to six minutes.[8] However, it is likely that in clinical practice patients have oxygen delivered through nebulisers for longer than this, particularly if two nebulisers are being given sequentially. We have shown that air-driven nebulisers will prevent the increase in $PaCO_2$ that results from use of oxygen-driven nebulisers in patients with stable COPD.[9] However, there are only two published RCTs in patients with acute exacerbations of COPD.[10 11] While acknowledging the important limitations of these studies, including lack of blinding, administration of single bronchodilator doses and low power, they did identify increased risk of an elevation in $PaCO_2$ in COPD patients with hypercapnia.[10 11]

The risks of oxygen delivered nebulisation in acute exacerbations of COPD need to be robustly defined to identify whether the widespread implementation of air driven

nebulisers is required to ensure the safe delivery of bronchodilators by nebulisation to this at-risk patient group.

2. Design & Objectives

A. DESIGN

A parallel group double blinded RCT comparing the effect of air versus oxygen driven bronchodilator nebulisation on $PaCO_2$, pH and oxygen saturation (SpO_2) in patients admitted to hospital with an acute exacerbation of COPD. Ninety patients will be recruited and randomised to receive two 15 minute administrations of salbutamol by nebulisation, delivered by air or oxygen at a flow rate of 8L/min.

B. OBJECTIVES

i. To compare the effects of air versus oxygen driven bronchodilator nebulisation on pCO_2, pH and SpO_2 in acute exacerbations of COPD
ii. To determine if the BTS recommendation of six minute oxygen driven nebuliser use avoids the risk of an oxygen induced increase in pCO_2

C. HYPOTHESES

i. Oxygen driven bronchodilator nebulisation results in increased pCO_2 in exacerbations of COPD compared with air driven bronchodilator nebulisation
ii. Oxygen driven bronchodilator nebulisation results in a reduction in pH in exacerbations of COPD compared with air-driven bronchodilator nebulisation
iii. The increase in pCO_2 and reduction in pH secondary to oxygen-driven nebuliser use are clinically significant in some patients, defined as $\geq$8mmHg change in $PaCO_2$ or $\geq$0.06 change in pH.
iv. Clinically significant elevations in pCO_2 secondary to oxygen-driven nebuliser use occurs within six minutes[11] of commencement of nebulisation in some patients
v. Cessation of oxygen-driven bronchodilator nebulisation may result in rebound hypoxaemia

3. Study Subjects

A. INCLUSION CRITERIA

i. Ninety patients admitted to Hutt Valley or Wellington Regional Hospital medical inpatient wards with a primary admission diagnosis of an exacerbation of COPD.
ii. Participants must have the mental capacity to allow them to provide written informed consent.

B. EXCLUSION CRITERIA

i. Age <40 years at time of randomisation
ii. Requirement for assisted non-invasive ventilation at time of randomisation
iii. Baseline $PtCO_2$ >60mmHg at time of randomisation
iv. Requirement for ≥4L/min of oxygen via nasal cannulae to maintain SpO_2 between 88-92% prior to or during titration
v. Any other condition which, at the investigator's discretion, is believed may present a safety risk or impact the feasibility of the study or the study results.

4. Randomisation and blinding

A. RANDOMISATION

Randomisation will be 1:1 via a block randomised (block size 6) computer generated sequence, provided by the study statistician independent of recruitment and assessment of participants. The allocated intervention will be stored in an opaque sealed envelope and opened at the time of randomisation by the un-blinded investigator.

B. BLINDING

i. Blinding of the blinded investigator during the study visit

The blinded investigator will be sat behind a portable screen so that they cannot see the participant or pulse oximeter screen. The participant will be asked not to

comment out loud whether they are wearing their mask or nasal prongs. The SpO_2 on the transcutaneous monitor will be covered.

ii. Blinding of the laboratory technician

The laboratory technician analysing the Capillary Blood Gas (CBG) sample will be masked to the randomised treatment.

iii. Blinding of the participant

The oxygen and air cylinders will not be distinguishable from each other to maintain blinding of the participant as to which gas their nebuliser is driven by. Participants will not be advised which treatment regimen they are randomised to or of the detail that one regimen involves removal of nasal prongs, if worn, (oxygen-driven) and the other does not (air driven). They will be informed, however, that their oxygen saturations will be monitored closely and if there are any concerns the regarding low oxygen saturation the un-blinded investigator will provide appropriate oxygen therapy immediately. Participants will not be able to see the screen of the pulse oximeter.

iv. Roles of the un-blinded investigator

a. Recording medical history, demographics and obtaining written informed consent (either investigator can do this prior to blinding).
b. CBG sampling
c. Randomisation
d. Administration of the randomised intervention
e. Recording and monitoring of finger probe SpO_2
f. Increase oxygen therapy if SpO_2 <85%.

v. Roles of the blinded investigator

a. Recording medical history, demographics and obtaining written informed consent (either investigator can do this prior to blinding).
b. Recording of $PtCO_2$
c. Recording of heart rate
d. Advise the cessation of the intervention should the $PtCO_2$ rise by $\geq$10mmHg from baseline.

5. Nebuliser equipment

a. Hudson RCI Micro Mist Nebuliser Masks (Hudson RCI, Durham, North Carolina, USA) will be used to deliver the air and oxygen driven nebulised bronchodilator.
b. Salbutamol 2.5 mg will be used for each 15 minute nebulisation
c. Oxygen and air to drive the nebulisation will be supplied in compressed portable cylinders (size D).

6. Study visit conduct

A. RECRUITMENT, TIMING AND CONSENT

Potentially eligible participants will be identified on the wards and invited to take part in the study. Patients can be recruited at any time during their medical admission; however the study visit should be timed to deliver the nebuliser regimen as close as possible to the time of the next prescribed bronchodilator dose. Written Informed consent will take place prior to any study specific procedures.

B. DEMOGRAPHIC DATA COLLECTION

i. The following will be collected on medical history

a. Primary reason for admission
b. Age and ethnicity
c. Pack year history
d. History of:
    a. Long term oral prednisone use
    b. Home oxygen use
    c. Home nebuliser use
    d. Previous hypercapnic respiratory failure
    e. Previous assisted ventilation
e. Medical comorbidities
f. Medication history
g. Current oxygen prescription (if any)

h. Date, time, dose and method (nebuliser, spacer or inhaler only) of last bronchodilator medication(s)
i. Date, time, dose and method (nebuliser, spacer or inhaler only) of the next prescribed bronchodilator medication(s)

ii. The following will be measured

a. Weight and height

NOTE: Should the participant be too unwell to take these measures, data may be collected from clinical records or patient history. The method of collecting this data (investigator measurement, clinical records or patient history must be documented).

b. In participants that are able to perform spirometry, this will be done using a using a Micro Spirometer (Micro Medical Ltd, Rochester, UK) to measure FEV_1 and FVC, and according to ATS/ ERS criteria.[14 15]

C. STUDY SET UP

i. Transcutaneous monitor calibration

The transcutaneous monitor screen displaying StO_2 is to be covered. The transcutaneous monitor will be attached to the participant's earlobe as per manufacturer's instructions. If the reading from the earlobe is inadequate, the forehead (over frontal bone) or clavicle can be used as back-up sites, as per the manufacturer's recommendations. A calibration period of at least 20 minutes will take place. This will be followed by measurements of $PtCO_2$ every 1 minute until consecutive measurements are within 1mmHg.

ii. Pulse oximeter application

The finger oximeter will then be placed on the participant's index finger. The StO_2 screen will be uncovered on the transcutaneous monitor and the un-blinded investigator will record the time, StO_2 and SpO_2. The transcutaneous monitor screen displaying StO_2 will be covered again and until t=80.

iii. **Blinded investigator set up**

From this point on and until t=80 minutes the blinded investigator and transcutaneous monitor will go behind a screen to avoid visualisation of the participant and pulse oximeter screen.

D. WASH IN PERIOD

i. **Baseline measures**

a. The un-blinded investigator will record the time, SpO_2 and nasal cannulae oxygen therapy flow (if any)
b. The blinded investigator will record the time, $PtCO_2$ and heart rate

ii. **Intervention**

For at least 15 minutes the un-blinded investigator will titrate oxygen therapy via nasal cannulae to achieve an SpO_2 of 88-92%. Note this may mean the participant breathes room air. If the participant requires $\geq$ 4L/min via nasal cannulae, they will be excluded.

iii. **Monitoring and measurements by the un-blinded investigator**

a. Every 5 minutes (to the nearest minute) record the time and SpO_2.
b. Monitor the SpO_2 and if the nasal cannulae oxygen flow is changed, document the time and new oxygen flow

iv. **Monitoring and measurements by the blinded investigator**

a. Every 5 minutes (to the nearest minute) record the time, $PtCO_2$ and heart rate.

E. RANDOMISED REGIMEN

i. **Baseline measures**

a. At the end of the wash in and immediately prior to the randomised regimen the un-blinded investigator will take a CBG, as per the SOP. On visualisation of blood entering the capillary tube they will ask the blinded investigator to record the time and $PtCO_2$.

b. Immediately after the CBG sampling, the following baseline (t=0 min) measurements will be made:
    i. The un-blinded investigator will record the time, SpO_2 and nasal cannulae oxygen therapy flow (if any).
    ii. The blinded investigator will record the time, $PtCO_2$ and heart rate

ii. Randomisation

The randomisation envelope will be opened by the un-blinded investigator and regimens applied as below.

iii. Regimens

<u>Air driven nebuliser regimen</u>

a. t=0-15 min: 2.5 mg salbutamol by nebulisation, delivered by air at a flow rate of 8L/min
b. t=15-20 min: Removal of nebuliser mask
c. t=20-35 min: 2.5 mg salbutamol by nebulisation, delivered by air at a flow rate of 8L/min.

The nebuliser is to continue running for the entire 15 minutes at t=0-15 min and t=20-35 min, even if the bronchodilator medication has dissipated.

Oxygen therapy is to be continued to be titrated by the un-blinded investigator using nasal cannulae (worn under the nebuliser mask at 0-15 and 20-35 min). Note that if the patient was not receiving oxygen before the intervention (was breathing room air) this will continue unless the oxygen saturations drop to <85%. If this occurs the nebuliser mask will be removed, nasal prongs attached and oxygen given before reattaching the nebuliser mask. If this does happen it will be recorded in the case report form.

<u>Oxygen driven nebuliser regimen</u>

a. t=0-15 min: 2.5 mg salbutamol by nebulisation, delivered by oxygen at a flow rate of 8L/min
b. t=15-20 min: Removal of nebuliser mask

c. t=20-35 min: 2.5 mg salbutamol by nebulisation, delivered by oxygen at a flow rate of 8L/min.

The nebuliser is to continue running for the entire 15 minutes at t=0-15 min and t=20-35 min, even if the bronchodilator medication has dissipated.

Any nasal cannulae oxygen therapy is to be discontinued and nasal prongs removed, during t=0-15 min and t=20-35 min. Oxygen must be restarted at the same flow given prior to nebuliser delivery (i.e. at t=15 the oxygen must be started at the flow delivered at t=0, and at t=35 it must be started again at the flow delivered at t=20 minutes). Note that if the patient was not receiving oxygen before the intervention (was breathing room air) this will continue unless the oxygen saturations drop to <85%. If this occurs the nebuliser mask will be removed, nasal prongs attached and oxygen given. If this does happen it will be recorded in the case report form.

iv. Regimen monitoring and measurements by the un-blinded investigator

a. At t= 5, 6, 10, 15, 20, 25, 26, 30, 35 record the time and SpO_2.
b. Monitor the SpO_2 and if the nasal cannulae oxygen flow is changed, document the time and new oxygen flow (air driven regimen only).
c. Document the time the nebulised medication canister becomes empty.
d. As close as possible prior to the completion of the second nebuliser at t=35 minutes, a CBG measurement will be taken, as per the SOP. On visualisation of blood entering the capillary tube un-blinded investigator ask blinded investigator to record the time and $PtCO_2$. The CBG will be taken whilst the air/oxygen-driven nebuliser is still in place: if the investigator runs into technical difficulties (for example, slow blood flow when collecting CBG sample), then the nebuliser shall be removed at no later than t=37 minutes.

NOTE: The t=20 measure is to occur immediately prior to initiation of the 2nd nebuliser and the t= 15 and t=35 measures are to occur immediately prior to cessation of nebuliser delivery. In the event of a $PtCO_2$ rise of ≥10mmHg

from baseline whereby a nebuliser has to be terminated early, the CBG measurement will be taken at this time-point. The top priority if this occurs is removal of oxygen and clinical assessment of the patient. CBG MUST OCCUR AFTER THIS.

v. Regimen monitoring and measurements by the blinded investigator

a. At t= 5, 6, 10, 15, 20, 25, 26, 30, 35 record the time, $PtCO_2$ and heart rate.

b. Continuously monitor $PtCO_2$, and should it increase by $\geq$10mmHg from baseline measure (t=0), indicate this to the un-blinded investigator so they can terminate the regimen intervention.

c. Record the time and $PtCO_2$ during arterialised earlobe blood gas sampling, as prompted by the un-blinded investigator.

NOTE: The t=20 measure is to occur immediately prior to initiation of the 2nd nebuliser and the t= 15 and t=35 measures are to occur immediately prior to cessation of nebuliser delivery

F. POST REGIMEN MONITORING

i. Intervention

The un-blinded investigator is to document the nasal prong oxygen flow instituted at the end of the second nebuliser.* Until t=80 oxygen will be delivered at this flow rate via nasal cannulae, except if the SpO_2 falls to <85%. In this case the flow is to be titrated until the 88-92% target saturation range is met.

* If participant has been on the air driven regimen, this will be the titrated flow at t=35. If participant has been on the oxygen driven nebuliser this will be the flow delivered via nasal prongs prior to the last nebulisation (t=20 min).

ii. Measurements and monitoring by the un-blinded investigator

a. At t= 40, 45, 50, 55, 60, 65, 70, 75, 80 record the time and SpO_2

b. Monitor the SpO_2 and if the nasal cannulae oxygen flow is changed, document the time and new oxygen flow

iii. Measurements and monitoring by the blinded investigator

a. At t= 40, 45, 50, 55, 60, 65, 70, 75, 80 record the time, $PtCO_2$ and heart rate.

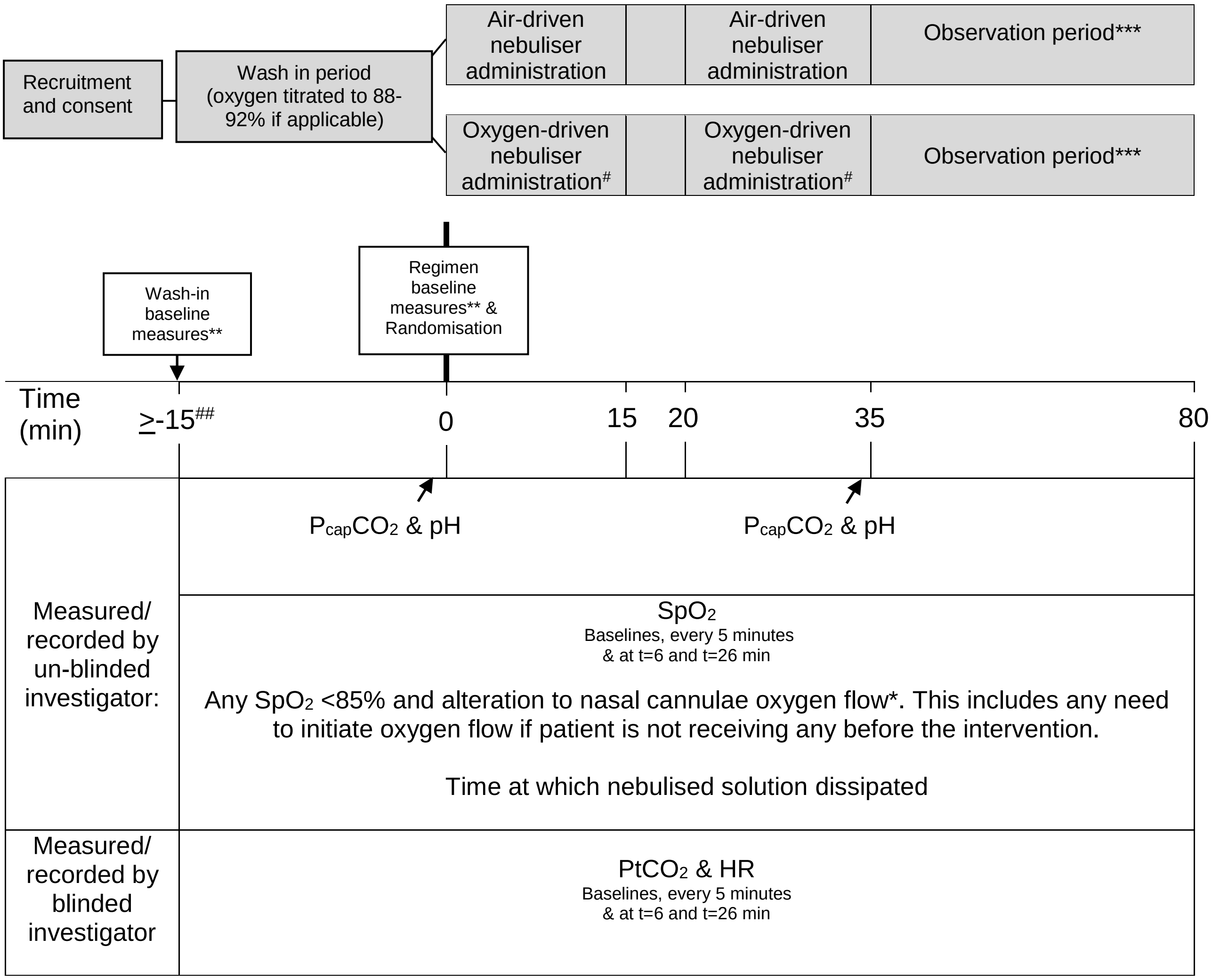

*From Time 0 only.

#Nasal cannulae oxygen delivery will be stopped during this time. It will be re-commenced at the time of the nebuliser finishing at the same rate as immediately before the nebuliser was given.

** Baseline measures are: Time; SpO_2; oxygen flow via nasal cannulae, if any; $PtCO_2$ and HR

Minimum time period of 15 minutes prior to capillary blood gas

*** Oxygen delivered at fixed flow (see section F.i.)

HR: Heart rate, $P_{cap}CO_2$: Capillary partial pressure of carbon dioxide, $PtCO_2$: Transcutaneous partial pressure of carbon dioxide, SpO_2: oxygen saturations

7. Outcome variables

A. COMPARATOR GROUPS

Outcome variables will be compared between the air versus oxygen driven nebuliser regimens.

B. OUTCOME MEASUREMENTS

Capillary blood gas samples (CBG) will measure pCO_2 ($P_{cap}CO_2$) and pH. The samples will be collected in capillary tubes and analysed at the Wellington Regional Hospital or Hutt Valley Hospital laboratory. The transcutaneous monitor will be placed on the participant's earlobe (or backup sites as listed above) to measure transcutaneous $PaCO_2$ ($PtCO_2$), oxygen saturation (StO_2) and heart rate. Finger pulse oximetry will measure oxygen saturation (SpO_2).

C. PRIMARY OUTCOME VARIABLE

i. P_tCO_2 at completion of the second nebulisation (at t=35min*), adjusted for baseline.
*or the last recorded measurement should the t=35 measurement not be obtained (e.g. study terminated early due to rise in $PtCO_2$ ≥10mmHg).

D. SECONDARY OUTCOME VARIABLES

i. $P_{cap}CO_2$ at completion of the second nebulisation (immediately prior to t=35min), adjusted for baseline $P_{cap}CO_2$.
ii. pH at completion of the second nebulisation (immediately prior to t=35 min), adjusted for baseline.
iii. $PtCO_2$ at six minutes after the initiation of the first and second nebulisers (t=6 min and t=26 min)
iv. $PtCO_2$, SpO_2 and heart rate at 5 minute intervals from t=0 to t=80 min

v. Greatest $PtCO_2$ change from baseline at any of the recorded time points between t=0 and t=35 minutes when the nebuliser is in place.
vi. Proportion of participants with $\geq$4mmHg (physiologically significant)[12 13] and $\geq$8mmHg (clinically significant)[12 13] increase in $P_{cap}CO_2$ or $PtCO_2$ from baseline
vii. Number of intervention terminations due to a rise in $PtCO_2$ $\geq$10mmHg
viii. The proportion of patients with a reduction in pH of ≥ 0.06 from baseline
ix. Proportion of patients who required initiation of an increase in the flow of oxygen therapy during the regimen and observation periods.

NOTE: $PtCO_2$ measurements recorded and analysed will be the actual (raw) data from the Transcutaneous Monitoring System, but adjustments for 'drift' will also be reported upon.

8. Methodology notes

A. WASH IN, REGIMEN AND OBSERVATION PERIODS

Delivery of two 15 minute nebulisers with a 5 minute gap with a 2.5mg salbutamol dose were selected to represent real-life nebuliser delivery in the acute setting. Titration of oxygen before and during the regimens represents recommended evidence based on best practice.[2 8]

Maintenance of any nasal cannulae oxygen therapy at a constant flow during the observation period is designed to assess the risk of rebound hypoxia (a reduction in SpO_2 following abrupt cessation of oxygen therapy to below the baseline level prior to instituting oxygen therapy). To detect any rebound hypoxemia a constant fraction of inspired oxygen is required. The results would represent the risk of rebound hypoxia in the situation that oxygen saturation monitoring does not occur following the abrupt cessation of high concentration oxygen therapy.

B. PRIMARY OUTCOME

Elevated pCO_2, is a potentially harmful physiological response to oxygen administration[2 3 8-11 16-20] and predictor of adverse clinical outcome.[21 22] This allows a

robust power calculation[9] and direct comparison to previous case and interventional studies that have investigated the use of oxygen driven nebulisers.[9-11]

C. CLINICALLY SIGNIFICANT OUTCOMES

A rise in $PtCO_2$ from baseline of $\geq$4mmHg is considered a physiologically significant change and $\geq$8mmHg a clinically significant change, based on previous definitions.[12] A change in pH $\geq$0.06 is based on the magnitude of the difference in pH that is expected to result from an increase in $PaCO_2$ and $\geq$8mmHg and has clinical relevance as a marker of requirement for NIV in an exacerbation of COPD.[23]

D. CAPILLARY BLOOD GAS SAMPLING

CBG allows accurate measurement of pCO_2[24] and pH,[24 25] and is a less invasive alternative to ABG measurement. Whilst the earlobe is the preferred site for pO_2 measurements, sampling blood from the fingertip or earlobe accurately reflects arterial pCO_2 and pH over a wide range of values.[24] Fingertip sampling by retractable lancets as used commonly for blood sugar measurements is more familiar to participants and tends to yield better blood flow than earlobes. pH is a valuable outcome measure as it is an independent predictor of death in exacerbations of COPD.[26-28] Fingertips will be used in the first instance. However, in the event of unsuccessful sampling from a fingertip (e.g. sample too small) or contraindication (e.g. if a participant has particularly thick/tough skin on examination), then the earlobe can be used as a back-up site.

E. TRANSCUTANEOUS MONITORING

The transcutaneous monitor provides continuous and non-invasive $PtCO_2$ monitoring. The accuracy of $PtCO_2$ monitoring has been shown in a variety of settings including in healthy subjects,[29] acute exacerbations of COPD,[30] sleep disorders,[31] critical illness,[32] and other patients.[33 34]

9. Safety Monitoring

There will be a Data Monitoring Safety Board comprising independent physicians, which will review data from any participants in which an SAE or rise in $PtCO_2$ of

$\geq$10mmHg occurred. This meeting will occur after 50% of participants have been recruited, or will be arranged sooner if 5 patients experience a $PtCO_2$ rise of $\geq$10mmHg. The meeting will also be brought forward should any investigator have safety concerns – such as several patients in succession experiencing $PtCO_2$ elevation, but not meeting the number of 5. This meeting will include experts independent from the study.

10. Power and Statistical Methods

A difference in $PaCO_2$ of 4 mmHg represents a physiologically significant change.[12][13] In our controlled study of oxygen versus air driven nebulisers in stable COPD the standard deviation of $PtCO_2$ was 5.5.[9] With 90% power and alpha of 5% this requires a total of 82 patients to detect a 4 mmHg difference. We anticipate a drop-out rate of <10% so our target recruitment is 90 patients. Analysis will be by intention to treat. Participants recruited will be stratified in to groups that were receiving oxygen immediately prior to randomisation or those that were not. Our primary analysis is ANCOVA with P_tCO_2 as the response variable, and randomised treatment and baseline P_tCO_2 as co-variates. For other continuous outcome variables we will also use similar ANCOVA. Exploratory analyses for $PtCO_2$, heart rate and SpO_2 taken at five minute intervals will be presented graphically and analysed by mixed linear models.

11. Note on changes to protocol since previous version

This is Version 2.0, which was not submitted in the initial application of ethics approval.

12. References

1. Martin P, Glasgow H, Patterson J. Chronic obstructive pulmonary disease (COPD): smoking remains the most important cause. *N Z Med J* 2005;118(1213):U1409.
2. Austin MA, Wills KE, Blizzard L, Walters EH, Wood-Baker R. Effect of high flow oxygen on mortality in chronic obstructive pulmonary disease patients in prehospital setting: randomised controlled trial. *BMJ* 2010;341:c5462.
3. Murphy R, Driscoll P, O'Driscoll R. Emergency oxygen therapy for the COPD patient. *Emerg Med J* 2001;18(5):333-9.
4. New A. Oxygen: kill or cure? Prehospital hyperoxia in the COPD patient. *Emerg Med J* 2006;23(2):144-6.
5. Austin SJ, Chan C. Oxygen as a driving gas for nebulisers: safe or dangerous? *Br Med J (Clin Res Ed)* 1984;288(6415):488.
6. Lim TK, Tan WC. Acute carbon dioxide narcosis during inhalational therapy with oxygen powered nebulizers in patients with chronic airflow limitation. *Ann Acad Med Singapore* 1985;14(3):439-41.
7. Hadfield JW, Stinchcombe SJ, Bateman JR. Oxygen as a driving gas for nebulisers: safe or dangerous? *Br Med J (Clin Res Ed)* 1984;288(6419):795.
8. O'Driscoll BR, Howard LS, Davison AG. BTS guideline for emergency oxygen use in adult patients. *Thorax* 2008;63 Suppl 6:vi1-68.
9. Edwards L, Perrin K, Williams M, Weatherall M, Beasley R. Randomised controlled crossover trial of the effect on PtCO2 of oxygen-driven versus air-driven nebulisers in severe chronic obstructive pulmonary disease. *Emerg Med J* 2012;29:894-898
10. Gunawardena KA, Patel B, Campbell IA, MacDonald JB, Smith AP. Oxygen as a driving gas for nebulisers: safe or dangerous? *Br Med J (Clin Res Ed)* 1984;288(6413):272-4.
11. O'Donnell D, Kelly CP, Cotter P, Clancy L. Use of oxygen driven nebulizer delivery systems for beta-2 agonists in chronic bronchitis. *Ir J Med Sci* 1985;154(5):198-200.
12. Wijesinghe M, Williams M, Perrin K, Weatherall M, Beasley R. The effect of supplemental oxygen on hypercapnia in subjects with obesity-associated hypoventilation: a randomized, crossover, clinical study. *Chest* 2011;139(5):1018-24.
13. Perrin K, Wijesinghe M, Healy B, Wadsworth K, Bowditch R, Bibby S, et al. Randomised controlled trial of high concentration versus titrated oxygen therapy in severe exacerbations of asthma. *Thorax* 2011;66(11):937-41.
14. Miller MR, Hankinson J, Brusasco V, Burgos F, Casaburi R, Coates A, et al. Standardisation of spirometry. *Eur Respir J* 2005;26(2):319-38.
15. Miller MR, Crapo R, Hankinson J, Brusasco V, Burgos F, Casaburi R, et al. General considerations for lung function testing. *Eur Respir J* 2005;26(1):153-61.
16. Prime FJ, Westlake EK. The respiratory response to CO2 in emphysema. *Clin Sci (Lond)* 1954;13(3):321-32.
17. Westlake EK, Simpson T, Kaye M. Carbon dioxide narcosis in emphysema. *Q J Med* 1955;24(94):155-73.

18. Campbell EJ. A method of controlled oxygen administration which reduces the risk of carbon-dioxide retention. *Lancet* 1960;2(7140):12-4.
19. Massaro DJ, Katz S, Luchsinger PC. Effect of various modes of oxygen administration on the arterial gas values in patients with respiratory acidosis. *Br Med J* 1962;2(5305):627-9.
20. Warrell DA, Edwards RH, Godfrey S, Jones NL. Effect of controlled oxygen therapy on arterial blood gases in acute respiratory failure. *Br Med J* 1970;1(5707):452-5.
21. Groenewegen KH, Schols AM, Wouters EF. Mortality and mortality-related factors after hospitalization for acute exacerbation of COPD. *Chest* 2003;124(2):459-67.
22. Soler-Cataluna JJ, Martinez-Garcia MA, Roman Sanchez P, Salcedo E, Navarro M, Ochando R. Severe acute exacerbations and mortality in patients with chronic obstructive pulmonary disease. *Thorax* 2005;60(11):925-31.
23. NICE. Chronic obstructive pulmonary disease. National clinical guideline on management of chronic obstructive pulmonary disease in adults in primary and secondary care. *Thorax* 2004;59 Suppl 1:1-232.
24. Zavorsky GS, Cao J, Mayo NE, Gabbay R, Murias JM. Arterial versus capillary blood gases: a meta-analysis. *Respir Physiol Neurobiol* 2007;155(3):268-79.
25. Murphy R, Thethy S, Raby S, Beckley J, Terrace J, Fiddler C, et al. Capillary blood gases in acute exacerbations of COPD. *Respir Med* 2006;100(4):682-6.
26. Denniston AK, O'Brien C, Stableforth D. The use of oxygen in acute exacerbations of chronic obstructive pulmonary disease: a prospective audit of pre-hospital and hospital emergency management. *Clin Med* 2002;2(5):449-51.
27. Roberts CM, Stone RA, Buckingham RJ, Pursey NA, Lowe D. Acidosis, non-invasive ventilation and mortality in hospitalised COPD exacerbations. *Thorax* 2011;66(1):43-8.
28. Plant PK, Owen JL, Elliott MW. One year period prevalence study of respiratory acidosis in acute exacerbations of COPD: implications for the provision of non-invasive ventilation and oxygen administration. *Thorax* 2000;55(7):550-4.
29. Fuke S, Miyamoto K, Ohira H, Ohira M, Odajima N, Nishimura M. Evaluation of transcutaneous CO2 responses following acute changes in PaCO2 in healthy subjects. *Respirology* 2009;14(3):436-42.
30. Cox M, Kemp R, Anwar S, Athey V, Aung T, Moloney ED. Non-invasive monitoring of CO2 levels in patients using NIV for AECOPD. *Thorax* 2006;61(4):363-4.
31. Senn O, Clarenbach CF, Kaplan V, Maggiorini M, Bloch KE. Monitoring carbon dioxide tension and arterial oxygen saturation by a single earlobe sensor in patients with critical illness or sleep apnea. *Chest* 2005;128(3):1291-6.
32. Rodriguez P, Lellouche F, Aboab J, Buisson CB, Brochard L. Transcutaneous arterial carbon dioxide pressure monitoring in critically ill adult patients. *Intensive Care Med* 2006;32(2):309-12.
33. McVicar J, Eager R. Validation study of a transcutaneous carbon dioxide monitor in patients in the emergency department. *Emerg Med J* 2009;26(5):344-6.
34. Perrin K, Wijesinghe M, Weatherall M, Beasley R. Assessing PaCO2 in acute respiratory disease: accuracy of a transcutaneous carbon dioxide device. *Intern Med J* 2011;41(8):630-3.
