# Supplementary material for: Oxygen versus air-driven nebulisers for exacerbations of chronic obstructive pulmonary disease: a randomised controlled trial
Source: BMC Pulm Med. 2018 Oct 3;18:157. doi: 10.1186/s12890-018-0720-7 (PMC6171193; doi:10.1186/s12890-018-0720-7)
Supplement: Supplementary file 3 — Online supplement - Table S1. PtCO2 change ≥4 mmHg according to randomised treatment. Table S2. SpO2 mixed linear model comparisons: Oxygen minus Air. Table S3. Heart Rate mixed linear model comparisons: Oxygen minus Air. (DOC 80 kb) [file 12890_2018_720_MOESM3_ESM.doc]

**Additional file**

**OXYGEN VERSUS AIR-DRIVEN NEBULISERS FOR EXACERBATIONS OF CHRONIC OBSTRUCTIVE PULMONARY DISEASE: A RANDOMISED CONTROLLED TRIAL**

^1,2^George Bardsley, ^1,2,3^Janine Pilcher, ^1,2,3^Steven McKinstry

^1,2^Philippa Shirtcliffe, ^2,4^James Berry, ^1,2^James Fingleton, ^4^Mark Weatherall, ^1,2,3^Richard Beasley

^1^Capital and Coast District Health Board, Wellington, New Zealand

^2^Medical Research Institute of New Zealand, Wellington, New Zealand

^3^Victoria University Wellington, Wellington, New Zealand

^4^Wellington School of Medicine & Health Sciences, University of Otago Wellington, Wellington, New Zealand

**Table S1:** **PtCO_2_ change ≥ 4 mmHg according to randomised treatment**

|  | **N/N (%)** | |  |  |
| --- | --- | --- | --- | --- |
| **Time** | **Air** | **Oxygen** | **Risk Difference (95% CI)** | **P-value** |
| 5 | 0/45 (0) | 4/45 (8.9) | 8.9% (0.57 to 17.2) | 0.02 |
| 6 | 0/45 (0) | 6/45 (13.3) | 13.3% (3.4 to 23.3) | 0.005 |
| 10 | 0/45 (0) | 9/45 (20.0) | 20.0% (8.3 to 31.7) | <0.001 |
| 15 | 0/45 (0) | 13/45 (28.9) | 28.9% (15.6 to 42.1) | <0.001 |
| 20 | 0/44 (0) | 0/45 (0) |  |  |
| 25 | 0/43 (0) | 7/45 (15.6) | 15.6% (5.0 to 26.1) | 0.003 |
| 26 | 0/43 (0) | 10/45 (22.2) | 22.2% (10.1 to 34.4) | <0.001 |
| 30 | 0/44 (0) | 11/45 (24.4) | 24.4% (11.9 to 37.0) | <0.001 |
| 35 | 0/44 (0) | 14/45 (31.1) | 31.1% (17.6 to 44.6) | <0.001 |
| **At any time point** | **0/45 (0)** | **18/45 (40.0)** | **40% (25.7 to 54.3)** | **<0.001** |

PtCO_2_: Transcutaneous partial pressure of carbon dioxide

**Table S2: SpO_2_ mixed linear model comparisons: Oxygen minus Air**

| Time | SpO_2_ Mean (SD) | | Oxygen minus air | P-value |
| --- | --- | --- | --- | --- |
|  | [N=45 for each unless specified] | | (95% CI) |  |
|  | Oxygen | Air |  |  |
| 0 | 92.6 (2.4) | 92.6 (2.3) |  |  |
| 5 | 97.6 (1.7) | 93.3 (2.4) | 4.29 (3.48 to 5.11) | <0.001 |
| 6 | 97.5 (1.7) | 93.0 (2.5) | 4.54 (3.73 to 5.35) | <0.001 |
| 10 | 97.6 (1.3) | 92.3 (2.7) | 5.29 (4.48 to 6.11) | <0.001 |
| 15 | 97.7 (1.1) | 92.6 (2.4) | 5.16 (4.35 to 5.97) | <0.001 |
| 20 | 94.0 (2.2) | 92.1 (3.7)* | 1.88 (1.06 to 2.69) | <0.001 |
| 25 | 97.5 (1.7) | 93.0 (2.3)* | 4.52 (3.71 to 5.34) | <0.001 |
| 26 | 97.6 (1.4) | 92.6 (2.6)* | 4.95 (4.14 to 5.77) | <0.001 |
| 30 | 97.6 (1.1) | 92.6 (2.5)* | 5.00 (4.18 to 5.81) | <0.001 |
| 35 | 97.6 (1.4) | 92.5 (2.4)* | 5.09 (4.27 to 5.91) | <0.001 |
| 40 | 94.1 (2.7)* | 92.5 (2.4)* | 1.71 (0.89 to 2.53) | <0.001 |
| 45 | 92.6 (2.8)* | 92.5 (2.5)* | 0.16 (-0.66 to 0.98) | 0.70 |
| 50 | 92.3 (2.9)* | 92.9 (2.6)** | -0.55 (-1.37 to 0.27) | 0.19 |
| 55 | 92.2 (2.6) | 92.4 (2.7)** | -0.18 (-1.00 to 0.64) | 0.67 |
| 60 | 91.9 (2.7) | 92.3 (2.6)** | -0.40 (-1.22 to 0.42) | 0.34 |
| 65 | 91.5 (2.7) | 92.5 (2.8)*** | -1.00 (-1.82 to -0.17) | 0.018 |
| 70 | 92.0 (2.7)* | 92.7 (2.5)** | -0.64 (-1.47 to 0.18) | 0.12 |
| 75 | 91.8 (3.0) | 92.4 (3.1)** | -0.60 (-1.42 to 0.22) | 0.15 |
| 80 | 91.5 (2.8)* | 92.7 (3.0)** | -1.22 (-2.04 to -0.39) | 0.004 |

*N=44; **N=43; ***N=42

SpO_2_: Oxygen saturation measured by pulse oximeter

**Table S3: Heart Rate mixed linear model comparisons: Oxygen minus Air**

| Time | Heart Rate Mean (SD) | | Oxygen minus air | P-value |
| --- | --- | --- | --- | --- |
|  | [N=45 for each unless specified] | | (95% CI) |  |
|  | Oxygen | Air |  |  |
| 0 | 89.6 (15.7) | 87.0 (16.0) |  |  |
| 5 | 86.5 (14.2) | 86.8 (17.3) | -1.80 (-4.76 to 1.16) | 0.23 |
| 6 | 86.5 (15.0) | 88.2 (17.6) | -2.69 (-5.65 to 0.28) | 0.075 |
| 10 | 86.9 (14.9) | 88.2 (18.0) | -4.20 (-7.16 to -1.24) | 0.006 |
| 15 | 91.3 (14.9) | 88.0 (18.3)* | -3.75 (-6.72 to -0.79) | 0.013 |
| 20 | 87.7 (16.5) | 88.3 (18.1)* | 0.43 (-2.54 to 3.41) | 0.77 |
| 25 | 86.9 (14.6) | 87.9 (18.1)* | -3.53 (-6.5 to -0.56) | 0.02 |
| 26 | 87.4 (14.6) | 88.2 (18.6)* | -3.85 (-6.83 to -0.88) | 0.011 |
| 30 | 87.7 (14.3) | 88.1 (17.4)* | -3.66 (-6.63 to -0.69) | 0.016 |
| 35 | 91.6 (14.0)* | 90.8 (18.7)* | -3.28 (-6.25 to -0.31) | 0.031 |
| 40 | 92.9 (13.5) | 91.1 (18.6)* | -2.07 (-5.05 to 0.91) | 0.17 |
| 45 | 93.7 (13.8) | 90.8 (18.5)* | -1.01 (-4.07 to 1.87) | 0.47 |
| 50 | 93.0 (13.3) | 90.2 (19.2)* | -0.01 (-2.98 to 2.97) | 0.99 |
| 55 | 92.1 (13.0) | 90.8 (17.2)* | -0.08 (-3.05 to 2.89) | 0.96 |
| 60 | 92.7 (14.2) | 91.7 (18.6)* | -1.54 (-4.51 to 1.43) | 0.31 |
| 65 | 92.1 (11.9)* | 91.6 (18.3)* | -1.92 (-4.89 to 1.06) | 0.21 |
| 70 | 92.9 (13.4) | 90.4 (16.8)* | -1.62 (-4.60 to 1.36) | 0.29 |
| 75 | 93.9 (13.5) | 90.7 (18.1)* | -0.38 (-3.35 to 2.60) | 0.80 |
| 80 | 87.2 (14.7) | 86.5 (16.4)* | 0.31 (-2.67 to 3.28) | 0.84 |

*N=44
